# An extended genotyping framework for *Salmonella enterica* serovar Typhi, the cause of human typhoid

**DOI:** 10.1038/ncomms12827

**Published:** 2016-10-05

**Authors:** Vanessa K. Wong, Stephen Baker, Thomas R. Connor, Derek Pickard, Andrew J. Page, Jayshree Dave, Niamh Murphy, Richard Holliman, Armine Sefton, Michael Millar, Zoe A. Dyson, Gordon Dougan, Kathryn E. Holt, International Typhoid Consortium, Julian Parkhill, Nicholas A. Feasey, Robert A. Kingsley, Nicholas R. Thomson, Jacqueline A. Keane, François- Xavier Weill, Simon Le Hello, Jane Hawkey, David J. Edwards, Simon R. Harris, Amy K. Cain, James Hadfield, Peter J. Hart, Nga Tran Vu Thieu, Elizabeth J. Klemm, Robert F. Breiman, Conall H. Watson, W. John Edmunds, Samuel Kariuki, Melita A. Gordon, Robert S. Heyderman, Chinyere Okoro, Jan Jacobs, Octavie Lunguya, Chisomo Msefula, Jose A. Chabalgoity, Mike Kama, Kylie Jenkins, Shanta Dutta, Florian Marks, Josefina Campos, Corinne Thompson, Stephen Obaro, Calman A. MacLennan, Christiane Dolecek, Karen H. Keddy, Anthony M. Smith, Christopher M. Parry, Abhilasha Karkey, Sabina Dongol, Buddha Basnyat, Amit Arjyal, E. Kim Mulholland, James I. Campbell, Muriel Dufour, Don Bandaranayake, Take N. Toleafoa, Shalini Pravin Singh, Mochammad Hatta, Paul N. Newton, David Dance, Viengmon Davong, Robert S. Onsare, Lupeoletalalelei Isaia, Guy Thwaites, Lalith Wijedoru, John A. Crump, Elizabeth De Pinna, Satheesh Nair, Eric J. Nilles, Duy Pham Thanh, Paul Turner, Sona Soeng, Mary Valcanis, Joan Powling, Karolina Dimovski, Geoff Hogg, Jeremy Farrar, Alison E. Mather, Ben Amos

**Affiliations:** 1 The Wellcome Trust Sanger Institute, Hinxton, Cambridge CB10 1SA, UK; 2 Addenbrooke's Hospital, Cambridge University Hospitals NHS Foundation Trust, Cambridge Biomedical Campus, Hills Road, Cambridge CB2 0QQ, UK; 3 The Hospital for Tropical Diseases, Wellcome Trust Major Overseas Programme, Oxford University Clinical Research Unit, Ho Chi Minh City, Vietnam; 4 Centre for Tropical Medicine and Global Health, Nuffield Department of Clinical Medicine, Oxford University, Oxford, UK; 5 Department of Infectious and Tropical Diseases, London School of Hygiene and Tropical Medicine, London WC1E 7HT, UK; 6 Cardiff University School of Biosciences, Cardiff University, Cardiff, UK; 7 Public Health Laboratory London, Public Health England, London, UK; 8 Division of Infection, Barts Health NHS Trust, London, UK; 9 Centre for Systems Genomics, University of Melbourne, Parkville, Victoria 3010, Australia; 10 Department of Biochemistry and Molecular Biology, Bio21 Molecular Science and Biotechnology Institute, University of Melbourne, Parkville, Victoria 3010, Australia; 11 Liverpool School of Tropical Medicine, Pembroke Place, Liverpool L3 5QA, UK; 12 Institute of Food Research, Norwich Research Park, Colney, Norwich NR4 7UA, UK; 13 Institut Pasteur, Unité des Bactéries Pathogènes Entériques, Paris, France; 14 Faculty of Veterinary and Agricultural Sciences, University of Melbourne, Parkville, Victoria 3052, Australia; 15 Institute of Biomedical Research, School of Immunity and Infection, College of Medicine and Dental Sciences, University of Birmingham, Birmingham, UK; 16 Kenya Medical Research Institute, PO Box 43640-00100, Nairobi, Kenya; 17 Centers for Disease Control and Prevention, 1600 Clifton Road, Atlanta, Georgia 30329-4027, USA; 18 Emory Global Health Institute, 1599 Clifton Road, NE 1599-001-1AH, Atlanta, Georgia 30322, USA; 19 Department of Infectious Disease Epidemiology, Centre for the Mathematical Modelling of Infectious Diseases, London School of Hygiene and Tropical Medicine, Keppel Street, London WC1E 7HT, UK; 20 Institute of Infection and Global Health, University of Liverpool, Liverpool L69 7BE, UK; 21 Malawi-Liverpool-Wellcome-Trust Clinical Research Programme, College of Medicine, University of Malawi, PO Box 30096, Blantyre 3, Chichiri, Malawi; 22 Division of Infection and Immunity, University College London, London, UK; 23 Department of Clinical Sciences, Institute of Tropical Medicine, Antwerp, Belgium; 24 Department of Microbiology and Immunology, KU Leuven, University of Leuven, Leuven, Belgium; 25 National Institute for Biomedical Research, Kinshasa, Democratic Republic of the Congo; 26 University Hospital of Kinshasa, Kinshasa, Kinshasa, Democratic Republic of the Congo; 27 Department of Microbiology, College of Medicine, University of Malawi, Zomba, Malawi; 28 Departamento de Desarrollo Biotecnologico, Instituto de Higiene, Facultad de Medicina, Avda A Navarro 3051, Montevideo CP 11600, Uruguay; 29 Ministry of Health, Toorak, PO Box 2223, Suva, Fiji; 30 Fiji Health Sector Support Program, PO Box 14986, Suva, Fiji; 31 National Institute of Cholera and Enteric Diseases, P-33 CIT Road, Scheme XM, Beliaghata, Kolkata 700 010, India; 32 Department of Epidemiology, International Vaccine Institute, Kwanak PO Box 14, Seoul 151-600, Republic of Korea; 33 Division of Enteropathogen, ANLIS-Carlos G Malbran Institute, CABA, Argentina; 34 Division of Pediatric Infectious Diseases, University of Nebraska Medical Center, Omaha, Nebraska 68198, USA; 35 University of Abuja Teaching Hospital, Gwagwalada, Federal Capital Territory, Nigeria; 36 Bingham University, Karu, Nassarawa State, Nigeria; 37 The Jenner Institute, Nuffield Department of Medicine, University of Oxford, Oxford, UK; 38 Division in the National Health Laboratory Service and Faculty of Health Sciences, Centre for Enteric Diseases, National Institute for Communicable Diseases, University of the Witwatersrand, Johannesburg, South Africa; 39 Department of Clinical Research, London School of Hygiene and Tropical Medicine, Keppel Street, WC1E 7HT, London, UK; 40 Graduate School of Tropical Medicine and Global Health, Nagasaki University, Nagasaki, Japan; 41 Patan Academy of Health Sciences, Wellcome Trust Major Overseas Programme, Oxford University Clinical Research Unit, Kathmandu, Nepal; 42 Murdoch Childrens Research Institute, Melbourne, Victoria, Australia; 43 Enteric and Leptospira Reference Laboratory, Institute of Environmental Science and Research Limited, New Zealand; 44 National Centre for Biosecurity and Infectious Disease, Institute of Environmental Science and Research, Porirua, New Zealand; 45 Samoa Ministry of Health, Apia, Samoa; 46 National Influenza Center, World Health Organization, Center for Communicable Disease Control, Suva, Fiji; 47 Department of Microbiology, Hasanuddin University, Makassar, Indonesia; 48 Lao-Oxford-Mahosot Hospital-Wellcome Trust Research Unit, Microbiology Laboratory, Mahosot Hospital, Vientiane, Lao People's Democratic Republic; 49 National Health Services, Tupua Tamasese Meaole Hospital, Apia, Samoa; 50 Mahidol-Oxford Tropical Medicine Research Unit, Faculty of Tropical Medicine, Mahidol University, Bangkok, Thailand; 51 Paediatric Emergency Medicine, Chelsea and Westminster Hospital, London, UK; 52 Centre for International Health, University of Otago, PO Box 56, Dunedin 9054, New Zealand; 53 Salmonella Reference Service, Public Health England, Colindale, London NW9 5EQ, UK; 54 Emerging Disease Surveillance and Response, Division of Pacific Technical Support, World Health Organization, PO Box 113, Suva, Fiji; 55 Cambodia-Oxford Medical Research Unit, Angkor Hospital for Children, Siem Reap, Cambodia; 56 Microbiological Diagnostic Unit—Public Health Laboratory, Department of Microbiology and Immunology at the Peter Doherty Institute for Infection and Immunity, The University of Melbourne, Melbourne, Victoria 3010, Australia; 57 Department of Veterinary Medicine, University of Cambridge, Cambridge, UK; 58 St Augustine's Hospital, Muheza, Tanzania

## Abstract

The population of *Salmonella enterica* serovar Typhi (*S.* Typhi), the causative agent of typhoid fever, exhibits limited DNA sequence variation, which complicates efforts to rationally discriminate individual isolates. Here we utilize data from whole-genome sequences (WGS) of nearly 2,000 isolates sourced from over 60 countries to generate a robust genotyping scheme that is phylogenetically informative and compatible with a range of assays. These data show that, with the exception of the rapidly disseminating H58 subclade (now designated genotype 4.3.1), the global *S*. Typhi population is highly structured and includes dozens of subclades that display geographical restriction. The genotyping approach presented here can be used to interrogate local *S.* Typhi populations and help identify recent introductions of *S*. Typhi into new or previously endemic locations, providing information on their likely geographical source. This approach can be used to classify clinical isolates and provides a universal framework for further experimental investigations.

## Abstract (web summary)

Typhoid fever is caused by *Salmonella enterica* serovar Typhi (S. Typhi). This study examines ∼2,000 clinical isolates of *S*. Typhi to show highly structured/geographically restricted genomes except rapidly disseminating H58 subclade, and design a genotyping framework for tracking the disease.

## Graphical abstract

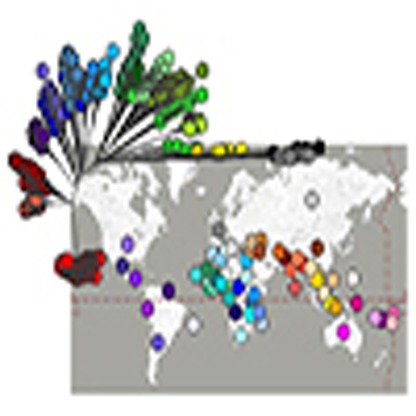

Typhoid fever (typhoid), caused by *Salmonella enterica* serovar Typhi (*S*. Typhi) bacteria, is a systemic human infection that affects an estimated 20.6 million people globally each year, causing an estimated 223,000 deaths^1^,^2^,^3^. Typhoid remains endemic in populations with limited access to sanitation and safe water, and is a notifiable or reportable infection in many industrialized countries, where it is generally associated with travel to endemic areas. Public health laboratories have relied on techniques such as phage typing^4^,^5^ or pulsed-field gel electrophoresis^6^, which are phylogenetically naive and have limited discriminatory power to support epidemiological investigations and surveillance.

A genotyping scheme based on 88 single-nucleotide polymorphisms (SNPs) identified within a limited set of genes was previously developed for *S*. Typhi^7^. This enabled the classification of the *S*. Typhi population into 85 haplotypes (haploid genotypes) based on biallelic profiles and provided the first phylogenetic framework for epidemiological studies^8^. Subsequently, whole-genome sequencing (WGS) has been used to identify many more SNPs and other phylogenetically informative markers for discriminating within *S*. Typhi, which has limited genetic variation^9^,^10^,^11^,^12^,^13^,^14^,^15^,^16^. Similar progress has been made in other monophyletic clades of bacterial pathogens, such as *Mycobacterium tuberculosis*^17^ and *Yersinia pestis*^18^.

We have recently reported the WGS of almost 2,000 *S*. Typhi isolates sourced from 63 countries^14^. This study identified >22,000 chromosomal SNPs in the core genome, which were used to build a comprehensive phylogenetic tree. Notably, the analysis confirmed the emerging dominance of the multidrug resistance-associated H58 clade, including the recent spread of H58 *S*. Typhi into Africa, confirming the value of SNP-based WGS analysis of *S*. Typhi to understand contemporary typhoid epidemiology. Here we utilize these WGS data to define a global population framework for *S*. Typhi and to define a new genotyping scheme comprising 68 SNPs that provides extensive coverage of typhoid-causing bacteria circulating globally. Given the increasingly widespread adoption of WGS by public health laboratories for the tracking of bacterial pathogens^19^,^20^, we further aimed to explore the utility of *S*. Typhi WGS data, analysed via genotyping, to predict the geographical source of travel-associated *S*. Typhi isolated in the United Kingdom. This approach gives greater discriminatory power and improved phylogenetic information than the earlier scheme^7^, and forms a robust framework for public health surveillance, epidemiological investigations and laboratory experiments of typhoid.

## Results

### Defining phylogenetically informative genotypes for *S*. Typhi

**Figure 1:**
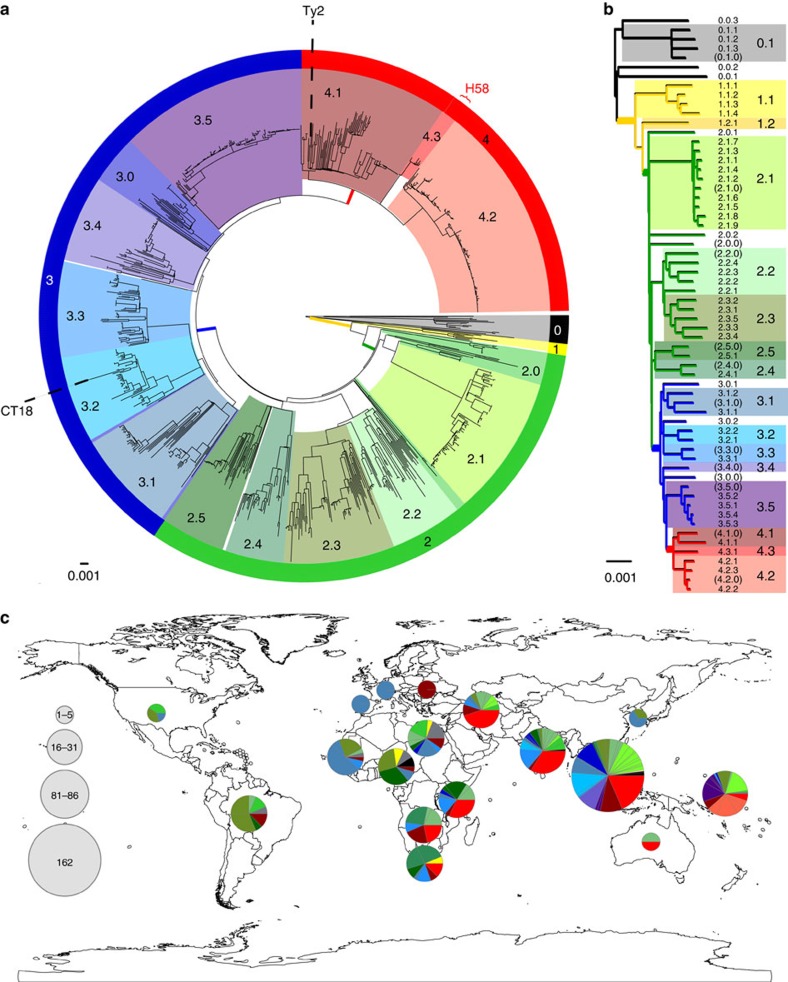
Population structure of *S*. Typhi based on genome-wide SNPs. (**a**) Whole-genome tree of 1,831 global *S*. Typhi isolates. Primary clusters 1–4 are indicated in the outer coloured ring; branches defining these groups are coloured in the tree. These groups are further divided into clades, which are shaded and labelled. The location of *S.* Typhi reference genomes CT18 (accession number AL513382) and Ty2 (accession number AE014613) are indicated on the tree. Subclade 4.3.1 (H58, marked in red), which comprises half of the global collection, is represented by just 50 (6%) randomly selected isolates out of the total 852 belonging to this subclade, so that the relationships between other clades can be visualized. (**b**) Tree backbone showing further division of 16 *S*. Typhi clades (shaded) into 49 subclades (labelled; note 12 undifferentiated clade groups shown in brackets). Branches are coloured by primary cluster. (**c**) Map of the world showing subclade diversity of *S*. Typhi isolates in the global collection, by region^22^. Where groups of isolates from the same country and year belonged to the same subclade, this was classified as an ‘outbreak' and the group is only represented once in the pie graphs. Pies are sized to indicate number of isolates; slices are coloured by clade; multiple slices of the same colour indicate multiple subclades belonging to the same clade.

In order to develop a comprehensive genotyping system, we used WGS data from >1,800 globally representative *S*. Typhi^14^ to identify phylogenetically informative clades and subclades based on SNP architecture^21^. A summary of the isolates is shown in Table 1 and full details are provided in Supplementary Data 1 and Supplementary Table 1. Using a combination of phylogenetic tree topology and population genetic methods (using BAPS; Bayesian Analysis of Population Structure^21^), we defined 16 *S*. Typhi clades that could be further divided into 49 subclades (Fig. 1, see Methods). Most of the clades could be grouped into four nested clusters (1–4, which we refer to as ‘primary clusters'), each with 100% bootstrap support and defined by >20 SNPs (coloured branches in Fig. 1a). The median pairwise distances between isolates were as follows: 25 SNPs within subclades, 109 SNPs within clades and 243 SNPs between clades. We labelled these primary clusters, clades and subclades using a structured hierarchical nomenclature system similar to that used for *M. tuberculosis*^17^, whereby cluster 1 is subdivided into clades 1.1 and 1.2; clade 1.1 is further subdivided into subclades 1.1.1, 1.1.2, 1.1.3 and so on (see Fig. 1b, Methods). An interactive version of the global phylogeny, with strains labelled by genotype, country of origin and year of isolation, is available at http://microreact.org/project/styphi^22^.

Under the new genotype nomenclature, the globally disseminated multidrug resistant clone commonly referred to as H58 (which actually includes haplotype H58 and eight other H58-derived haplotypes under the original Roumagnac *et al*. scheme^7^), constitutes a single subclade (4.3.1). No other subclades were identified within clade 4.3. The CT18 reference genome (H1 in Roumagnac *et al*. scheme) belonged to subclade 3.2.1, while the laboratory strain Ty2 and its attenuated mutant BRD948 (H10 under the Roumagnac scheme) belonged to clade 4.1 (with no further differentiation to subclade level by BAPS). The backbone of the minimum spanning tree of Roumagnac *et al*. haplotypes was broadly consistent with the backbone structure of the whole-genome phylogeny (Supplementary Fig. 1a). However, mapping the Roumagnac haplotypes to the whole-genome phylogeny showed that the older scheme provides highly uneven resolution across the *S*. Typhi phylogeny (Supplementary Fig. 1b), with a lack of resolution in some cases (11 Roumagnac haplotypes span two or more distinct subclades each; for example, H52 comprises clades 3.4, 3.5, 4.1 and 4.2) and excessive resolution in others (24 subclades are further divided into two or more haplotypes in the Roumagnac scheme).

### A new SNP-based genotyping framework for *S.* Typhi

We identified a minimum set of 68 SNPs that can be used to genotype *S*. Typhi into the four primary clusters, 16 clades and 49 subclades. For each of these groups, we identified all SNPs that were unique to members of the group, and selected one such SNP to be used for genotyping. We prioritized the inclusion of synonymous intragenic SNPs (that is, located within a protein-coding sequence, but with no change to the encoded amino acid), within genes that showed evidence of genetic stability within the *S*. Typhi population (that is, nucleotide diversity <1% and d*N*/d*S* <0.7 across the global data set, with no inactivating mutations identified). Details of the genotyping SNPs are given in Supplementary Table 2. This genotyping scheme has greater discriminatory power than the original Roumagnac haplotyping scheme (*D*=0.96 versus 0.78), is phylogenetically informative by design and the hierarchical nomenclature of genotypes is intrinsically informative with respect to phylogenetic relationships between clades and subclades.

### Geographical distribution of *S*. Typhi clades and subclades

Next, we examined the geographical distribution of *S*. Typhi genotypes. For these analyses, isolates of the same subclade, country and year were collapsed to a single representative to reduce the impact of localized outbreaks on our collection; this resulted in 541 unique isolates for analysis. Primary clusters 2, 3 and 4 were broadly distributed across continents (greens, blues and reds, respectively, in Fig. 1c), likely reflecting the relatively ancient spread of *S*. Typhi across the globe. Isolates outside these clusters, which result from deep branching closer to the root of the *S*. Typhi whole-genome tree, were rare in our collection (*n*=24 unique isolates) and mostly found in Africa (*n*=16). While the three common clusters (2–4) were present in most regions we analysed, cluster 2 predominated among American isolates (*n*=18/23 unique isolates, 78%). Most clades were detected on multiple continents (*n*=11/16) and included isolates from Asia (*n*=13/16) and/or Africa (*n*=10/16), which together made up 78% of our isolate collection (Table 1). However, there were differences in the geographic distributions of clades, with most clades being dominated by unique isolates from a single continent (Asia, Africa or Oceania; see Supplementary Fig. 2).

**Figure 2:**
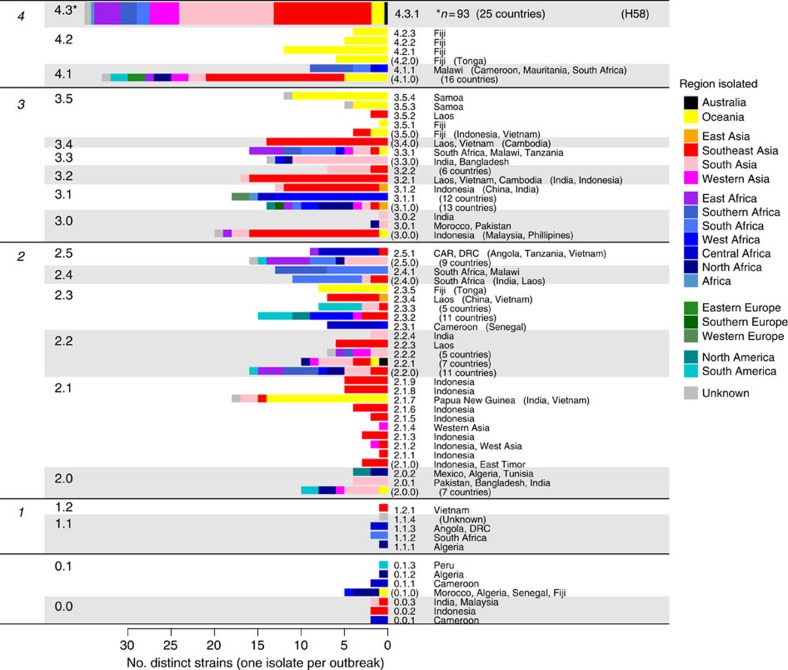
Geographical clustering of *S.* Typhi subclades. Heatmap shows, for each subclade, the percentage of unique isolates originating from each of the geographical regions. Where groups of isolates from the same country and year belonged to the same subclade, this was classified as an ‘outbreak' and the group is only represented once. The same data are represented as a scaled bar graph to the right. The full list of isolates by country and subclade is provided in Supplementary Data 1.

In contrast, at the subclade level, only 22% of subclades (*n*=11) were found on more than one continent, and most were dominated by unique isolates from a single country or region: 40 subclades (82%) had ⩾50% of non-outbreak isolates from a single country (Fig. 2) and 44 subclades (90%) had ⩾50% of non-outbreak isolates from a single region (Fig. 2). A total of 28 subclades comprised five or more non-outbreak isolates each, and of these common subclades, 12 (43%) were detected in a single region only (six in Oceania, five in Southeast Asia and one in South Asia; Fig. 2). In total, 16 common subclades (57%) were highly restricted to a region (>90% of isolates drawn from a single region) and 20 (71%) were generally associated with one region (>70% of isolates drawn from a single region; Fig. 2). These data suggest that most *S*. Typhi subclades represent localized bacterial subpopulations with barriers to geographical dispersion, and that transfers to new locations rarely result in long-term establishment of local populations. In contrast with this general pattern, subclade 4.3.1 (previously H58) was found in nine different regions across Africa, Asia and Oceania. Only 10 other subclades (20%) were found on more than one continent, and the majority of these were dominated either by Asian, African or Oceanian isolates (Fig. 2). Thus, the recent global dissemination of subclade 4.3.1, which spread out of South Asia ∼30 years ago and has established successful local clonal expansions in dozens of countries^14^, likely represents a comparatively rare event in the evolutionary history of *S*. Typhi.

### Genomic prediction of the geographical origins of *S.* Typhi by comparison with the global framework

Since most *S*. Typhi subclades were associated with a narrow geographical source, we hypothesized that genotyping of *S*. Typhi isolates could be used to predict the likely geographical origins of typhoid cases. As this is clearly challenging for the more widely distributed subclades, we also sought to examine whether specific SNPs could be used to predict origins down to the country level. For 1,501 out of 1,831 (82%) isolates in our global collection, the genetically closest isolate was from the same country. Where the closest isolate was 0–1 SNPs away, this frequency was 95% and for <10 SNPs, 90% (Supplementary Fig. 3).

**Figure 3:**
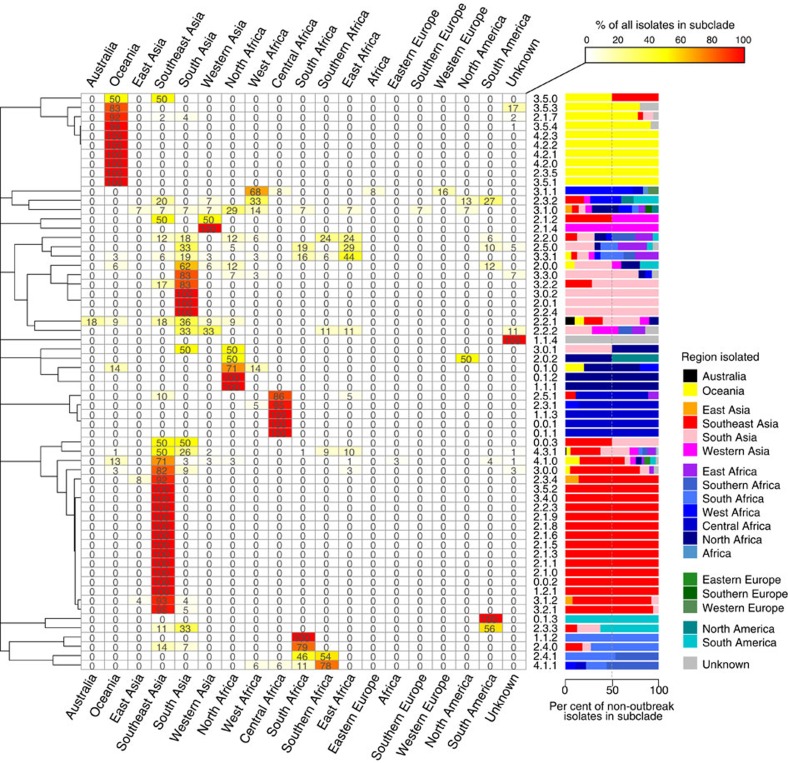
Phylogeny of 99 travel-associated *S*. Typhi in comparison with the global genomic framework containing 1,831 isolates. Whole-genome SNP tree is shown in the centre and branches are coloured by clade. Rings indicate region of origin: inner ring, global collection; outer ring, travel-associated isolates. Subclades that contain travel-associated isolates are highlighted within the tree (shaded in alternating colours) and labelled around the outside; intrasubclade phylogenies are provided in Supplementary Fig. 4.

Since our current global genome collection includes groups of isolates that were frequently collected from the same time and place, this should not be taken as a reliable measure of the general predictive power of SNP distance for *S*. Typhi. In order to further explore the power of our global genomic framework to predict geographic origins of travel-associated typhoid, we sequenced and genotyped 99 novel *S*. Typhi that were isolated from patients attending a hospital in East London, United Kingdom between 2005 and 2010 (Table 2). A total of 13 genotypes were identified. Epidemiological interviews were able to link 81 of these cases with travel to a specific country; the remaining 18 cases were not associated with travel. The median SNP distance between these novel isolates and genomes in our global collection was 21 SNPs (interquartile range, 18–25 SNPs), posing a challenge for prediction of their geographical origin. Among the 81 travel-associated UK isolates, 53 were genotyped as 4.3.1; these were all linked to travel to countries within South Asia (Table 3), and clustered along with South Asian isolates from the global collection (Fig. 3 and Supplementary Fig. 4n). For the 28 non-4.3.1 travel-associated UK isolates, the location of travel generally matched the geographical origin of the closest isolate (in terms of number of SNPs) in the global collection: travel location and closest global isolate source matched at the region level in all cases, and at the country level in most cases (*n*=20, 71%). That is, prediction of geographical origin based on the closest strain of known location in the current global framework would have yielded the correct region of origin in all cases, and the correct country of origin in 71% of cases (95% confidence interval (CI), 66–76%). Furthermore, for non-4.3.1 subclades, genotyping alone was predictive of geographical origin at the regional level for the same proportion of isolates (71%).

It is likely that power to predict the geographical sources of UK isolates would be improved by wider geographical coverage in the reference genome collection. Two of these isolates were genotyped as subclade 3.1.1 and linked with travel to Ghana and Nigeria; the closest isolates in our global collection were 16–17 SNPs away and were not from these precise locations, but likely originated from bordering countries in West Africa (Supplementary Fig. 4e). It is likely that a deeper coverage of West African isolates in our global framework would provide greater power to resolve geographic associations within this region, which comprised less than 2% of our current global collection (*n*=30 isolates). Similarly, for the other travel-associated isolates for which the recorded country of travel did not match the closest genome in the global tree, the closest genome was also from a neighbouring country (for example, Pakistan, India, Bangladesh; see Supplementary Fig. 4).

Genomic predictions of the geographical origins of 18 non-travel-associated UK isolates are shown in Table 4 and Supplementary Fig. 4. Thirteen isolates were 4.3.1 and clustered together with travel-associated isolates from South Asia, within a broader group of South Asian 4.3.1 isolates (Fig. 3). This suggests that *S*. Typhi imported into the United Kingdom from these regions have likely been transmitted onwards within the United Kingdom to individuals with no recent travel history (Supplementary Fig. 4n). Two additional isolates were from subclades that were dominated by a single region in our global collection—3.1.1 (68% West Africa) and 3.3.0 (83% South Asia). Notably, while the 4.3.1 isolates were closely related to travel-associated isolates recently obtained in London, they were ⩾17 SNPs away from any isolates in the global collection. Thus, the diversity captured by the global collection does not provide the resolution to precisely identify the origin of these isolates^23^.

## Discussion

Our data show that the global *S*. Typhi population consists of 49 distinct subclades that are strongly geographically clustered, with many locations harbouring subpopulations of *S*. Typhi established over long periods of time. We show how these subclades can be identified through a simple genotyping scheme consisting of 68 SNPs. Importantly, while we show that this scheme is highly phylogenetically informative, it can be readily inferred from raw sequence data without the need for multiple genome comparisons, phylogenetic analysis or any other complex or computationally intensive steps. Such properties make this universal SNP-based system a valuable tool upon which researchers can develop future studies. The *S.* Typhi genome is highly stable and exhibits minimal genetic variation and virtually no recombination^9^,^14^, and we recently estimated the substitution rate to be slower than one SNP per genome per year^14^; therefore, the genotyping framework is expected to be robust to future evolution.

Owing to the strong geographical clustering of the various subclades, whole-genome comparison of novel *S*. Typhi isolates to the existing global population framework is strongly predictive of geographic origin at the regional level and has the potential to accurately predict origins to the country level. This has important public health implications for typhoid surveillance and control in endemic and non-endemic areas; however, ongoing updates to the global genomic framework will be important to ensure the utility of genomic surveillance for typhoid. For example, we found that the origin of travel-associated 4.3.1 isolates could not be resolved using the prior global framework alone, but benefitted from updated information provided by other recent travel-associated isolates of known geographical origin. This illustrates the importance of expanding and updating the global genomic framework through sequencing of novel isolates and suggests that, while ongoing surveillance in endemic areas is undoubtedly important, the use of clinically well-characterized travel-associated organisms isolated in non-endemic countries may also provide a valuable source for improving the granularity of data in the framework for genome-based surveillance of *S*. Typhi^23^. In addition, it will be important to expand the current global framework to include more recent isolates (the most recent in our current collection was from 2013) as well as isolates from regions that are currently under-represented (including Africa, the Americas and northeast Asia).

WGS-equipped reference laboratories provide a highly accessible source to expand the global genomic framework for typhoid, with potential benefits to local but also global typhoid control. For example, in England, Wales and Northern Ireland ∼520 typhoid cases are reported annually to the national reference laboratory (Public Health England). These cases are investigated in order to determine whether they are associated with travel to typhoid endemic regions^23^. However, approximately one-fifth of typhoid cases in the United Kingdom cannot be traced to a country of origin. At present, Public Health England provides molecular typing, which since April 2015 includes WGS as well as antimicrobial susceptibility profiling, for *S*. Typhi isolated from such cases. The resulting data are considered important for local epidemiology. However, we propose that this could also serve as a proxy for informal surveillance of typhoid molecular epidemiology in endemic regions. This may prove particularly valuable when supported by our genotyping framework for simplified attribution.

## Methods

### Bacterial isolates and WGS

A total of 1,930 *S*. Typhi isolates were analysed in this study (Supplementary Data 1), including a collection of 1,831 globally distributed isolates contributed by members of the International Typhoid Consortium^14^ and 99 novel *S*. Typhi isolated in East London, UK. *S*. Typhi comprising the global collection were isolated between 1905 and 2013 and originate from 65 countries spanning six continents (Asia, Africa, North and South America, Europe, and Australia and Oceania) as previously described^14^.

An additional 99 novel *S*. Typhi isolates were obtained from returning travellers with a febrile illness who presented at The Royal London Hospital, Barts Health NHS Trust in East London, UK, between 2005 and 2012. Travel history, available for 81 of the travellers, included visits to seven countries within the continents of Asia and Africa. DNA was extracted using the Wizard Genomic DNA Kit (Promega, Madison, WI, USA) as per the manufacturer's instructions. Index-tagged paired end Illumina sequencing libraries were prepared as previously described^24^. These were combined into pools, each containing 96 uniquely tagged libraries, and were sequenced on the Illumina Hiseq2500 platform (Illumina, San Diego, CA, USA) according to the manufacturer's protocols to generate tagged 100 base pair (bp) paired-end reads.

### SNP analysis

For analysis of SNPs, the paired-end reads were mapped to the reference genome of *S.* Typhi CT18 (ref. 25), using SMALT (version 0.7.4; http://www.sanger.ac.uk/resources/software/smalt/). SNPs were identified as previously described^14^, using *samtools mpileup*^26^ and filtering with a minimum mapping quality of 30 and a quality ratio cutoff of 0.75 (ref. 24). SNPs located within phage regions, repetitive sequences or recombinant regions were excluded as previously described^14^, resulting in a final set of 22,143 chromosomal SNPs in an alignment length of 4,275,037 bp for the global collection of 1,831 *S*. Typhi isolates. An expanded alignment comprising 22,673 SNPs from *S.* Typhi isolates from the global collection (1,831) plus 99 traveller-associated UK isolates was generated using the same procedures as above. Pairwise SNP distances between isolates (that is, the number of core genome SNP loci at which pairs of isolates had discordant alleles) were extracted from each alignment *i* using the *ape* package^27^ for R (v3.2; function call: *dist.dna(i,model="N",pairwise.deletion=T)*).

### Phylogenetic analyses

The maximum likelihood (ML) phylogenetic tree shown in Fig. 1 was built from the 22,143-SNP alignment of all 1,831 isolates using RAxML (version 7.8.6)^28^ with the generalized time-reversible model and a Gamma distribution to model site-specific rate variation (the GTR+γ substitution model; GTRGAMMA in RAxML). The tree was outgroup-rooted by including a pseudo-sequence comprising *S. Paratyphi* A alleles in the alignment. Support for the ML phylogeny was assessed via 100 bootstrap pseudo-analyses of the alignment data.

The backbone topology of the global ML tree, showing relationships between subclades (Fig. 1b), was recovered by randomly selecting one isolate from each subclade to retain, and removing all other tips from the tree (using *drop.tips()* in the *ape* package^27^ for R (v3.2)). A ML phylogenetic tree was also generated separately from 22,673 SNPs of *S.* Typhi isolates from the global collection (1,831) plus the 99 East London traveller-associated isolates, using the same procedures as above. All ML trees were visualized and annotated using Python (https://github.com/katholt/plotTree/#python-code).

### Identification of phylogenetically informative clades and subclades

In addition to the whole-genome phylogenetic analysis outlined above, we investigated the population structure of the global *S*. Typhi collection using a phylogeny-free population genetics approach, implemented in BAPS v.6.0 (ref. 21). Hierarchal clustering analyses were conducted on identified clusters until single-member clusters were obtained, thus allowing the discovery of nested genetic population structures^21^. Ten nested levels of molecular variation were fitted to the data using 10 independent runs of the stochastic optimization algorithm with the *a priori* upper bound of the number of clusters varying over the interval 50–300 across the runs^30^.

As our goal was to identify genotypes that were both phylogenetically and epidemiologically informative, we explored the homogeneity (1-Simpson's diversity) of geographical source within BAPS clusters (as an indicator of the potential power of genotyping to identify geographical origin of travel-associated isolates) at different levels of clustering (Supplementary Fig. 5). This showed that within-cluster homogeneity increased up to the sixth level of clustering and then reached a plateau, with deeper clustering providing no greater resolution of geographical origin (Supplementary Fig. 5). The third level of clustering resulted in most clusters being dominated by a single continent (14/17 clusters with >80% of isolates from one continent), while sixth-level clustering resulted in most clusters containing isolates from a single country (60/89 clusters with >80% of isolates from one country; Supplementary Fig. 6). We therefore used the BAPS clusters to guide the definition of clades (BAPS level 3) and subclades (BAPS level 6).

In order to maintain compatibility with the phylogeny, some minor modifications of the raw BAPS clusters were required (this consisted of subdividing some BAPS clusters and merging others, but not reassigning members between clusters; see Supplementary Fig. 7). The modified level-3 BAPS clusters were designated ‘clades' and were assigned labels of the form [x].[y], where [x] indicates to which major cluster each clade belongs and [y] designates sister clades within each major cluster. The modified level-6 BAPS clusters were designated ‘subclades' and assigned labels of the form [x].[y].[z], where [x].[y] indicate to which clade each subclade belongs and [z] designates sister subclades within each clade. Thus, genotype names indicate relationships between genotypes; for example, 2.1.1 and 2.1.2 are sister subclades within clade 2.1, while 2.2.1 is a member of the distinct clade 2.2.

Some BAPS clusters were polyphyletic and consisted of isolates belonging to rare phylogenetic lineages whose common ancestor in the phylogenetic tree coincided with the common ancestor of an entire clade (*n*=9) or primary cluster (*n*=2). These groups contain isolates that, given increased numbers, may emerge as distinct BAPS clusters that form sister taxa within the parent clade (or primary cluster), and were thus designated [z]=0 (or [y]=0) to indicate non-equivalence with the properly differentiated sister clades (*n*=16) or subclades (*n*=49). For example, while the genotypes 2.1 and 2.2 represent distinct sister clades that are each monophyletic, isolates assigned to 2.0 are paraphyletic and include multiple lineages that could not be further subdivided by BAPS analysis (Supplementary Fig. 7).

Subclade 4.3.1, which is the only subclade of Clade 4.3, corresponds to the group referred to as H58, based on the haplotyping scheme of Roumagnac *et al*. in which it is defined by the presence of a single SNP *glpA*-C1047T (position 2,348,902 in *S*. Typhi CT18, BiP33 (ref. 7)). BAPS clustering at any level could not further subdivide subclade 4.3.1 (H58).

### SNP-based genotyping

We identified a minimum set of 68 SNPs with which to rapidly genotype *S*. Typhi into the 16 clades and 49 subclades, as described above (Supplementary Table 2). Short read alignment (BAM) files, generated by mapping Illumina reads to the CT18 reference genome (accession AL513382), were used to assign genotypes for each novel read set using a custom Python script (available at https://github.com/katholt/genotyphi). Briefly, the script uses *samtools mpileup* to extract from each BAM file the consensus base calls at the SNP loci. The resulting variant call format file is then processed to identify the presence of cluster-, clade- and/or subclade-defining SNP alleles (defined in Supplementary Table 2) that pass a minimum quality threshold (default consensus base Phred score ⩾20) and uses these to assign the read set to a cluster, clade and subclade. Discriminatory power was calculated using the method outlined in ref. 31.

### Data availability

Raw sequence data are available in the European Nucleotide Archive under accession ERP001718. Supplementary Data 1 lists accession numbers for each isolate. The software for Microreact interactive tree viewer is available at: http://microreact.org/project/styphi^22^. SMALT is available at: http://www.sanger.ac.uk/resources/software/smalt/. Python script to visualize and annotate trees is available at https://github.com/katholt/plotTree/#python-code. Python script to call SNPs is downloadable at https://github.com/katholt/genotyphi.

## Disclaimer

The findings and conclusions contained within this publication are those of the authors and do not necessarily reflect positions or policies of the Bill and Melinda Gates Foundation, and the content do not necessarily represent the official views of the National Institutes of Health. The funders had no role in study design, data collection and analysis, decision to publish or preparation of the manuscript.

## Additional information

**How to cite this article:** Wong, V.K. *et al*. An extended genotyping framework for *Salmonella enterica* serovar Typhi, the cause of human typhoid. *Nat. Commun.* 7:12827 doi: 10.1038/ncomms12827 (2016).

## Supplementary Material

Supplementary Information Supplementary Figures 1-7, Supplementary Tables 1-2 and Supplementary References.

Supplementary Dataset

## Figures and Tables

**Table 1:** Summary of 1,831 *S*. Typhi.

**Continent (*****region)***	**Country of origin (*****n*****⩾5)**	**Range of isolation dates (years)**	**Number of isolates**
*Asia*		*1972–2012*	*1068 (58.3%)*
Southeast Asia		1976–2012	719
	Cambodia	2007–2012	210
	Indonesia	1976–2012	129
	Laos	2000–2010	138
	Vietnam	1972–2011	221
	Malaysia	2005–2011	6
	Other (Philippines, Thailand, East Timor, Myanmar)	2002–2012	15
South Asia		1977–2012	321
	Bangladesh	1998–2012	51
	India	1977–2012	174
	Nepal	1999–2012	47
	Pakistan	2003–2012	45
	Other (Sri Lanka, Afghanistan)	2001–2012	4
Western Asia		1997–2011	25
	Iraq	2006–2011	11
	Lebanon	2001–2011	7
	Other (Armenia, Palestine, Turkey, Western Asia)	1997–2011	7
Eastern Asia		2002–2011	3
	Other (China)	2002–2011	3
			
*Africa*		*1958–2013*	*374 (20.4%)*
North Africa		1961–2009	24
	Algeria	1999–2009	7
	Morocco	1999–2000	9
	Other (Sudan, Egypt, Tunisia)	1961–2008	8
East Africa		1980–2010	115
	Kenya	1998–2009	56
	Tanzania	2006–2010	52
	Other (Comoros, Madagascar)	1980–2002	7
Central Africa		1958–2011	49
	Cameroon	1958–2009	27
	DRC	1976–2011	17
	Other (Angola, Central African Republic)	2001–2009	5
West Africa		1998–2009	30
	Other (Burkina Faso, Cape Verde, Benin, Guinea, Ivory Coast, Gabon, Liberia, Mali, Niger, Nigeria, Mauritania, Senegal, Togo)	1998–2009	30
Southern Africa		2004–2013	153
	Malawi	2004–2013	112
	South Africa	2004–2012	41
Africa		2009–2012	3
	Unknown	2009–2012	3
			
*Europe*		*1916–2009*	*7 (0.4%)*
Eastern Europe		1916–1996	2
	Other (Russia)	1916–1996	2
Western Europe		2009	4
	Other (France (suspected African origin of infection))	2009	4
Southern Europe		2009	1
	Other (Malta)	2009	1
			
*Australia and Oceania*		*1980–2012*	*342 (18.7%)*
Australia		2010–2012	3
	Australia	2010–2012	3
Oceania		1980–2012	339
	Fiji	1981–2012	170
	Samoa	1992–2012	117
	Papua New Guinea	1980–2012	47
	Other (Tonga, Vanuatu)	1980–2003	5
			
North America		1958–2011	5 (0.3%)
	Other (USA, Mexico)	1958–2011	5
			
Central America		2012	1 (0.05%)
	Other (El Salvador)	2012	1
			
South America		1905–2012	17 (0.9%)
	Argentina	1905–2006	10
	Other (French Guiana, Peru, South America )	2002–2012	6
Unknown origin		1939–2012	19 (1.0%)

*S*. Typhi, *Salmonella enterica* serovar Typhi.

Typhi isolates from the global collection, which were used to define genotypes. Countries with fewer than five isolates were grouped into the category ‘Other'; *n* indicates the number of such countries in each region.

**Table 2:** Summary of 99 East London travel-associated *S*. Typhi isolates used in the study.

**Country of origin**	**Range of isolation dates (year)**	**Number of isolates**
Bangladesh	2006–2012	38
India	2006–2012	22
Pakistan	2006–2012	13
Nepal	2007	1
India/Pakistan*	2008	1
India/Kuwait*	2008	2
Bangladesh/India*	2010	2
Nigeria	2009	1
Ghana	2007	1
No known travel	2005–2011	18
Total	2005–2012	99

*S*. Typhi, *Salmonella enterica* serovar Typhi.

The country of origin and range of isolation dates (years) for the isolates are described.

^*^For five patients, multiple countries of travel were recorded, and it was not possible to confirm in which country the *S*. Typhi infection originated.

**Table 3:** Summary of genotyping and SNP results for travel-associated *S.* Typhi isolates with known country of travel.

**Subclade**	**Country of travel**	***N*** **(travel)**	**Closest genome country match**	***N*** **(global)**	**Region frequencies (excluding outbreaks)**
**2.0.1**	Bangladesh	1	1	9	*South Asia (100%)
**2.1.7**	India	2	2	49	Oceania (92%)
**2.2.0**	Pakistan	1	1	17	Southern Africa (24%)East Africa (24%)South Asia (18%)
**2.2.2**	India	1	1	9	South Asia (33%)Western Asia (33%)
**2.3.3**	Bangladesh	2	2	9	South America (56%)South Asia (33%)
**3.1.1**	Nigeria, Ghana	2	0	25	*West Africa (68%)
**3.2.2**	Bangladesh, Pakistan	3	2	12	*South Asia (83%)
**3.3.0**	Bangladesh, Pakistan, India	14	13	30	*South Asia (83%)
**3.3.1**	Pakistan	1	1	32	East Africa (44%)South Asia (19%)
**4.1.0**	India	1	1	78	Southeast Asia (71%)
**4.3.1**	Bangladesh, India, Pakistan, Nepal, Kuwait	53	—	853	Southeast Asia (50%)South Asia (26%)

Closest genome country match, number of travel-associated isolates whose country of travel matched that of the closest genome in the global collection (based on lowest number of SNPs); *N* (global), number of isolates in the global collection that were assigned to this subclade; *N* (travel), number of travel-associated isolates that were assigned to the subclade; Region frequencies, frequency of each geographic region among isolates of this subclade from the global collection (note groups of isolates from the same subclade, country and year were classified as outbreaks and represented only once per group in the frequency calculations); SNP, single-nucleotide polymorphism; *S*. Typhi, *Salmonella enterica* serovar Typhi.

^*^Highlights the most frequent region for this subclade among the global collection, where this matches the region of travel.

**Table 4:** Summary of genotyping and SNP results for travel-associated *S.* Typhi isolates of unknown origin.

**Isolate**	**Country of closest SNP match**	**Distance (#SNPs)**	**Subclade**	**Subclade distribution**
H06434426	Mexico	146	2.0.2	North America (50%)North Africa (50%)
H06156550	Pakistan	92	3.0.1	South Asia (50%)North Africa (50%)
H05272442	Ghana	17	3.1.1	*West Africa (68%)
H09176223	Bangladesh	11	3.3.0	*South Asia (83%)
H10182335	India	18	3.3.1	East Africa (44%)South Asia (19%)
H10046338	Bangladesh†	13	4.3.1	Southeast Asia (50%)South Asia (26%)
H05406403	Bangladesh†	4		
H06136379	Bangladesh†	19		
H06136380	Bangladesh†	17		
H05118260	Bangladesh/India†	19		
H10382491	India†	9		
H10394694	India†	9		
H06016481	India†	9		
H05196407	India†	14		
H05196408	India†	12		
H05212226	India†	17		
H11372598	Pakistan†	10		
H09266336	Pakistan†	9		

SNP, single-nucleotide polymorphism; *S*. Typhi, *Salmonella enterica* serovar Typhi.

For each London isolate, the closest isolate in the global collection was determined (closest=smallest SNP distance, that is, smallest number of core genome SNPs); the country and SNP distance are recorded.

^*^Highlights the most frequent region for this subclade among the global collection, where this matches the region of the closest isolate in the global collection.

^†^Location of closest travel-associated isolates from London (unresolvable beyond ‘South Asia' based on the global collection alone).

## References

[b1] Parry C. M., Hien T. T., Dougan G., White N. J. & Farrar J. J. Typhoid fever. N. Engl. J. Med. 347, 1770–1782 (2002). 12456854 10.1056/NEJMra020201

[b2] Crump J. A. & Mintz E. D. Global trends in typhoid and paratyphoid Fever. Clin. Infect. Dis. 50, 241–246 (2010). 20014951 10.1086/649541 PMC2798017

[b3] Mogasale V. . Burden of typhoid fever in low-income and middle-income countries: a systematic, literature-based update with risk-factor adjustment. Lancet Global Health 2, e570–e580 (2014). 25304633 10.1016/S2214-109X(14)70301-8

[b4] Sen B. . Phage typing, biotyping & antimicrobial resistance profile of *Salmonella enterica* serotype Typhi from Kolkata. Indian J. Med. Res. 125, 685–688 (2007). 17642505

[b5] Kim S. . Clustering analysis of *Salmonella enterica* serovar Typhi isolates in Korea by PFGE, ribotying, and phage typing. Foodborne Pathog. Dis. 6, 733–738 (2009). 19580446 10.1089/fpd.2008.0212

[b6] Dutta S. . Antimicrobial resistance, virulence profiles and molecular subtypes of *Salmonella enterica* serovars Typhi and Paratyphi A blood isolates from Kolkata, India during 2009-2013. PLoS ONE 9, e101347 (2014). 25098613 10.1371/journal.pone.0101347 PMC4123848

[b7] Roumagnac P. . Evolutionary history of *Salmonella Typhi*. Science 314, 1301–1304 (2006). 17124322 10.1126/science.1134933 PMC2652035

[b8] Baker S. . High-throughput genotyping of *Salmonella enterica* serovar Typhi allowing geographical assignment of haplotypes and pathotypes within an urban District of Jakarta, Indonesia. J. Clin. Microbiol. 46, 1741–1746 (2008). 18322069 10.1128/JCM.02249-07 PMC2395080

[b9] Holt K. E. . High-throughput sequencing provides insights into genome variation and evolution in *Salmonella* Typhi. Nat. Genet. 40, 987–993 (2008). 18660809 10.1038/ng.195 PMC2652037

[b10] Holt K. E. . Temporal fluctuation of multidrug resistant *Salmonella* Typhi haplotypes in the Mekong river delta region of Vietnam. PLoS Negl. Trop. Dis. 5, e929 (2011). 21245916 10.1371/journal.pntd.0000929 PMC3014949

[b11] Holt K. E. . High-throughput bacterial SNP typing identifies distinct clusters of *Salmonella* Typhi causing typhoid in Nepalese children. BMC Infect. Dis. 10, 144 (2010). 20509974 10.1186/1471-2334-10-144 PMC2897797

[b12] Baker S. . Combined high-resolution genotyping and geospatial analysis reveals modes of endemic urban typhoid fever transmission. Open Biol. 1, 110008 (2011). 22645647 10.1098/rsob.110008 PMC3352080

[b13] Holt K. E. . Emergence of a globally dominant IncHI1 plasmid type associated with multiple drug resistant typhoid. PLoS Negl. Trop. Dis. 5, e1245 (2011). 21811646 10.1371/journal.pntd.0001245 PMC3139670

[b14] Wong V. K. . Phylogeographical analysis of the dominant multidrug-resistant H58 clade of *Salmonella* Typhi identifies inter- and intracontinental transmission events. Nat. Genet. 47, 632–639 (2015). 25961941 10.1038/ng.3281 PMC4921243

[b15] Pham Thanh D. . Identification of *Salmonella enterica* serovar Typhi genotypes by use of rapid multiplex ligation-dependent probe amplification. J. Clin. Microbiol. 51, 2950–2958 (2013). 23824765 10.1128/JCM.01010-13 PMC3754622

[b16] Baltazar M. . Multidrug-resistant *Salmonella enterica* serotype Typhi, Gulf of Guinea Region, Africa. Emerg, Infect, Dis. 21, 655–659 (2015). 25811307 10.3201/eid2104.141355 PMC4378479

[b17] Coll F. . A robust SNP barcode for typing *Mycobacterium tuberculosis* complex strains. Nat. Commun. 5, 4812 (2014). 25176035 10.1038/ncomms5812 PMC4166679

[b18] Morelli G. . *Yersinia pestis* genome sequencing identifies patterns of global phylogenetic diversity. Nat. Genet. 42, 1140–1143 (2010). 21037571 10.1038/ng.705 PMC2999892

[b19] Holmes A. . The utility of whole genome sequencing of *Escherichia coli* O157 for outbreak detection and epidemiological surveillance. J. Clin. Microbiol. 53, 3565–3573 (2015). 26354815 10.1128/JCM.01066-15 PMC4609728

[b20] Gardy J. L. . Whole-genome sequencing and social-network analysis of a tuberculosis outbreak. N. Engl. J. Med. 364, 730–739 (2011). 21345102 10.1056/NEJMoa1003176

[b21] Corander J., Marttinen P., Siren J. & Tang J. Enhanced Bayesian modelling in BAPS software for learning genetic structures of populations. BMC Bioinformatics 9, 539 (2008). 19087322 10.1186/1471-2105-9-539 PMC2629778

[b22] Aanensen D. M. . Whole-genome sequencing for routine pathogen surveillance in public health: a population snapshot of invasive *Staphylococcus aureus* in Europe. MBio 7, (2016). 10.1128/mBio.00444-16 PMC4959656 27150362

[b23] Public Health England. *Health Protection Report: Enteric fever surveillance quarterly report (England, Wales and Northern Ireland): first quarter 2015 Infection reports*, Volume 9 Number 16, Published on 8 May 2015.

[b24] Croucher N. J. . Rapid pneumococcal evolution in response to clinical interventions. Science 331, 430–434 (2011). 21273480 10.1126/science.1198545 PMC3648787

[b25] Parkhill J. . Complete genome sequence of a multiple drug resistant *Salmonella enterica* serovar Typhi CT18. Nature 413, 848–852 (2001). 11677608 10.1038/35101607

[b26] Li H. . The sequence alignment/map format and SAMtools. Bioinformatics 25, 2078–2079 (2009). 19505943 10.1093/bioinformatics/btp352 PMC2723002

[b27] Paradis E., Claude J. & Strimmer K. APE: analyses of phylogenetics and evolution in R language. Bioinformatics 20, 289–290 (2004). 14734327 10.1093/bioinformatics/btg412

[b28] Stamatakis A. RAxML-VI-HPC: maximum likelihood-based phylogenetic analyses with thousands of taxa and mixed models. Bioinformatics 22, 2688–2690 (2006). 16928733 10.1093/bioinformatics/btl446

[b29] Corander J. & Tang J. Bayesian analysis of population structure based on linked molecular information. Math Biosci. 205, 19–31 (2007). 17087977 10.1016/j.mbs.2006.09.015

[b30] Casali N. . Evolution and transmission of drug-resistant tuberculosis in a Russian population. Nat. Genet. 46, 279–286 (2014). 24464101 10.1038/ng.2878 PMC3939361

[b31] Hunter P. R. Reproducibility and indices of discriminatory power of microbial typing methods. J. Clin. Microbiol. 28, 1903–1905 (1990). 2229371 10.1128/jcm.28.9.1903-1905.1990 PMC268075

