# Supplementary material for: An extended genotyping framework for Salmonella enterica serovar Typhi, the cause of human typhoid
Source: Nat Commun. 2016 Oct 5;7:12827. doi: 10.1038/ncomms12827 (PMC5059462; doi:10.1038/ncomms12827)
Supplement: Supplementary Information — Supplementary Figures 1-7, Supplementary Tables 1-2 and Supplementary References. [file ncomms12827-s1.pdf]

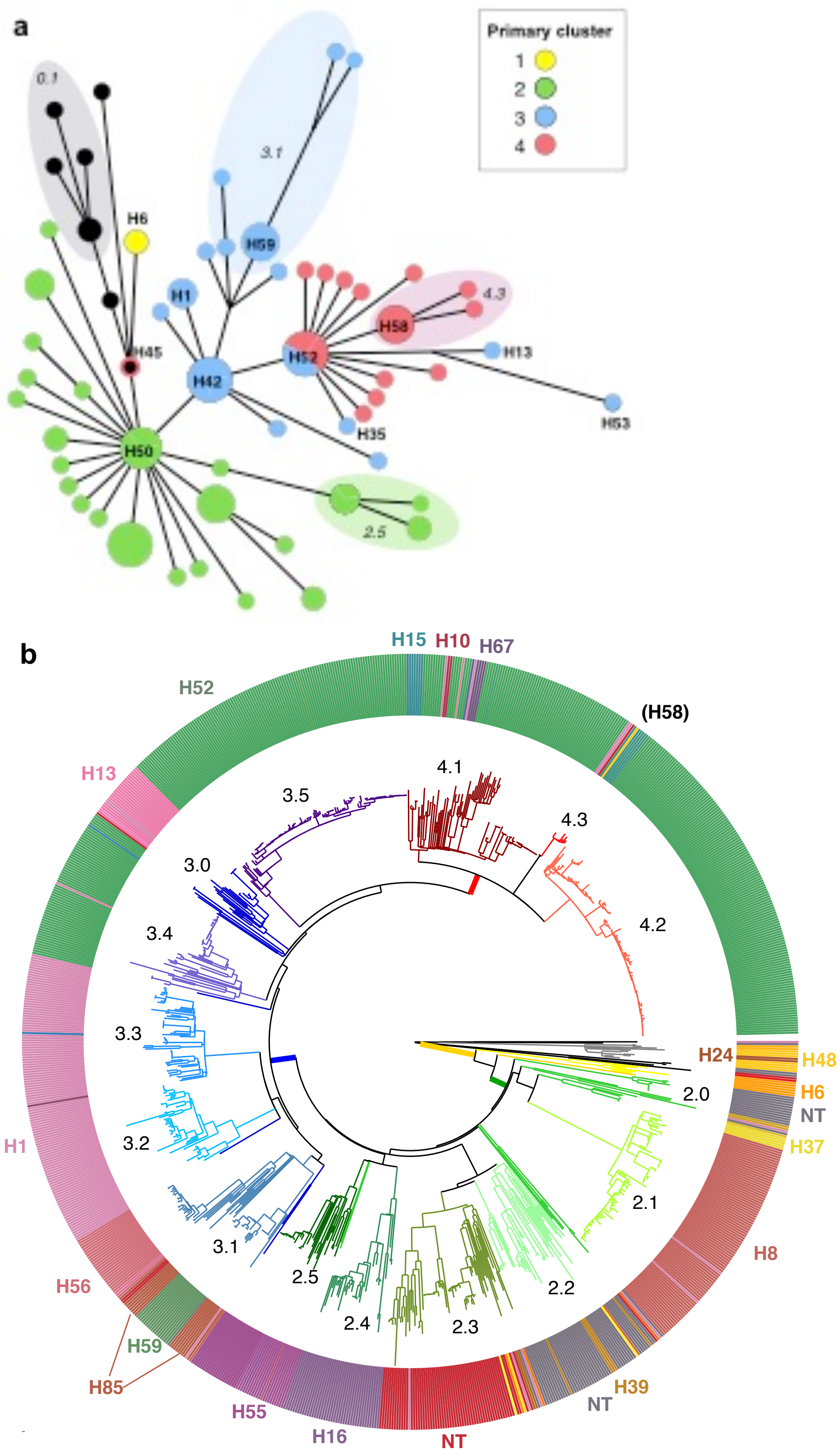

Supplementary Figure 1. Whole genome phylogeny for *S.* Typhi global collection in relation to the Roumagnac *et al.* haplotyping scheme [1]. (a) Roumagnac's global population structure of 105 *S.* Typhi based on SNPs in 199 gene fragments (88,739 base pairs) [1]. The tree shows 59 haplotypes (nodes) based on 88 alternative allelic states (biallelic polymorphisms; BiPS) with the four primary clusters (1-4) indicated by colors. Nodes that fall within the Clades 0.1, 2.5, 3.1 and

4.3 are highlighted by color ovals. H45 represents the ancestral root node (black circle with red border). **(b)** Whole genome phylogeny for *S*. Typhi global collection, showing haplotypes based on Roumagnac scheme and new clade genotypes. Tree shows maximum likelihood phylogeny for the global collection, based on genome-wide SNP calls. Branches are colored and labeled by clade as defined in this study. Outer colored ring shows 85 haplotypes in randomly assigned colors, using the scheme proposed in Roumagnac *et al*, 2006 [1]. Major haplotypes are labeled. NT = non-typeable.

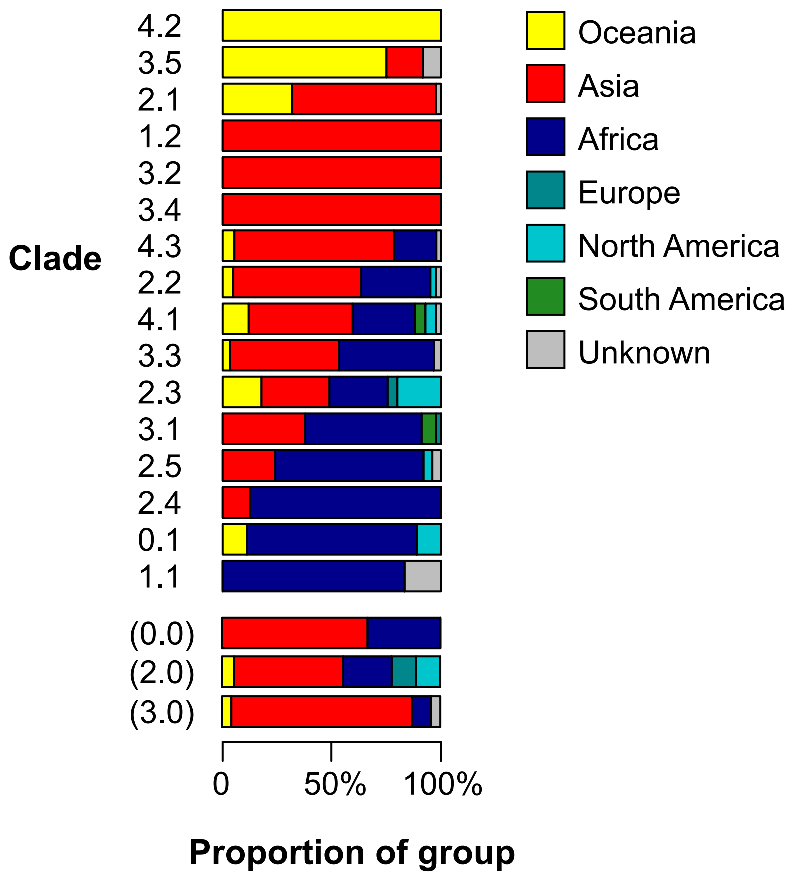

Supplementary Figure 2. Distribution of each clade by continent.
S. Typhi isolates of the same subclade, country and year were collapsed into a single representative, to reduce the impact of localized outbreaks on our collection (n=541 non-outbreak isolates). In addition to the 16 clades, the geographical distribution of isolates assigned to the cluster level but not further differentiated into clades are also shown (labeled in brackets).

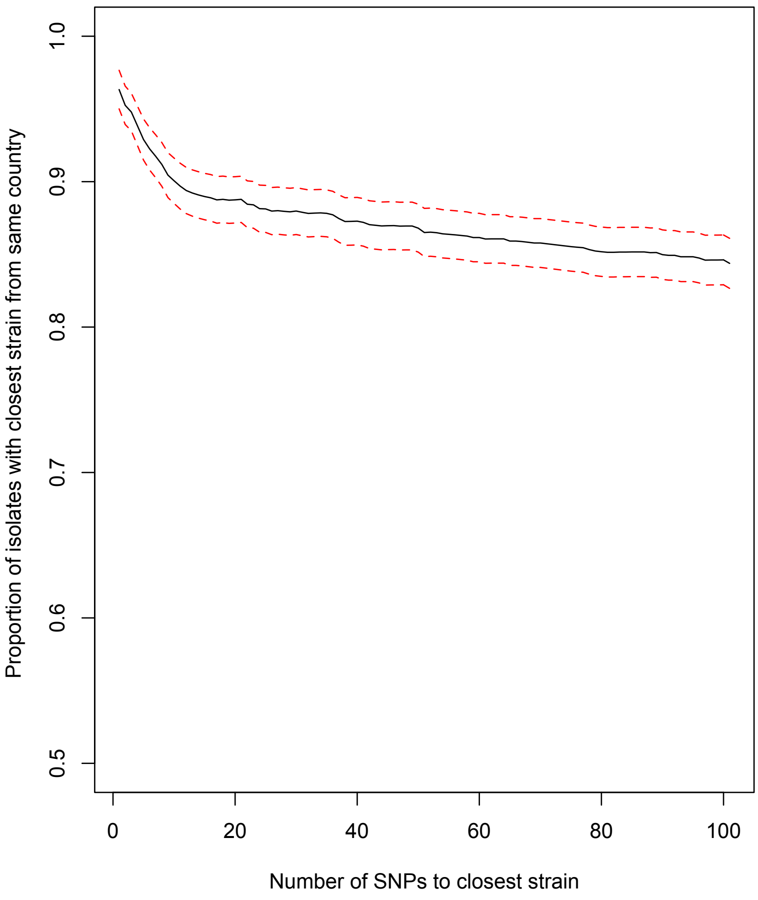

Supplementary Figure 3. Frequency of country matches between closest related isolates. Relationship between the proportion of *S*. Typhi isolates with closest strain from same country and the SNP distance to the closest strain. Solid black line = mean value; red dashed line = 95% confidence interval.

Supplementary Figure 4.

(a) Genotype 4.1.0

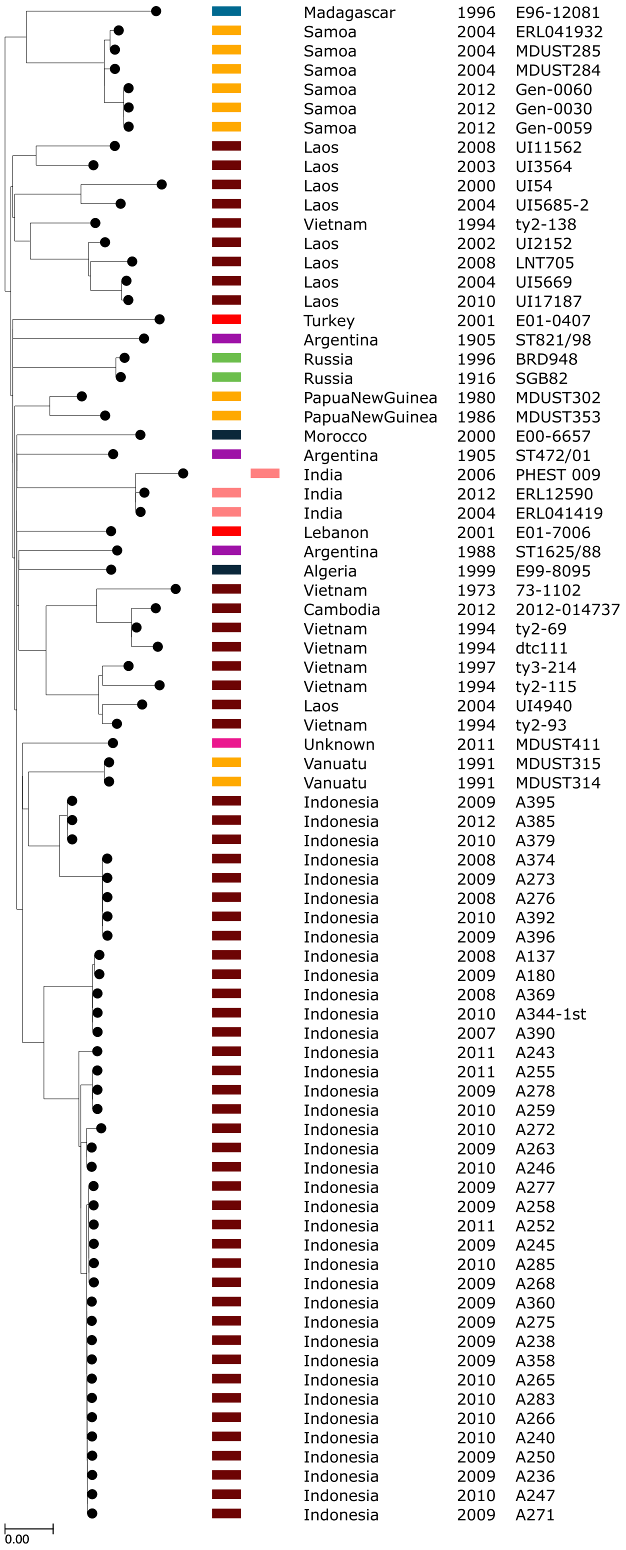

(b) Subclade 3.3.1

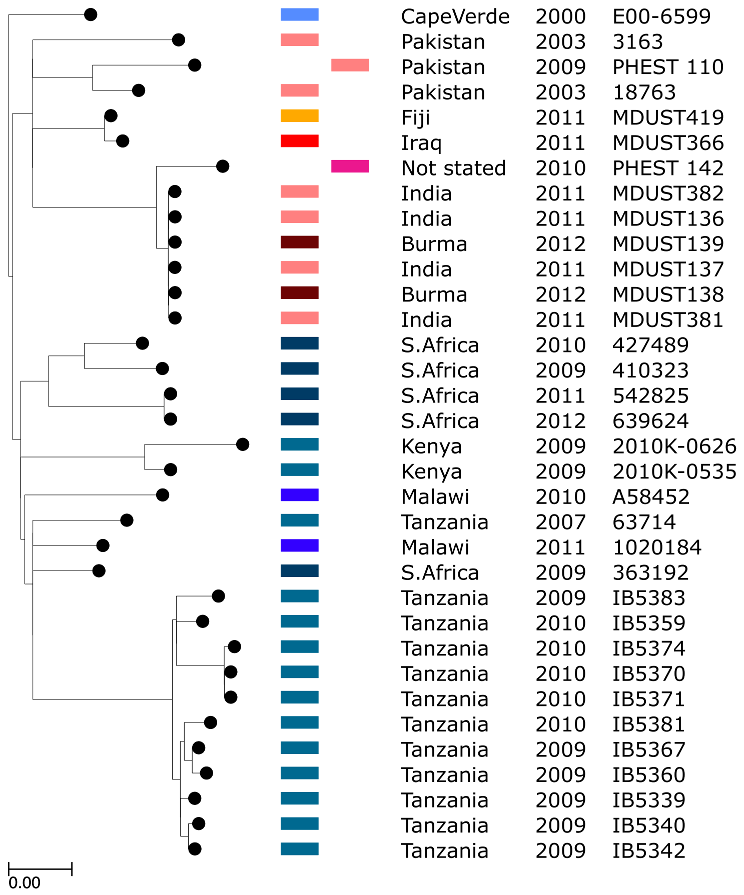

(c) Genotype 3.3.0

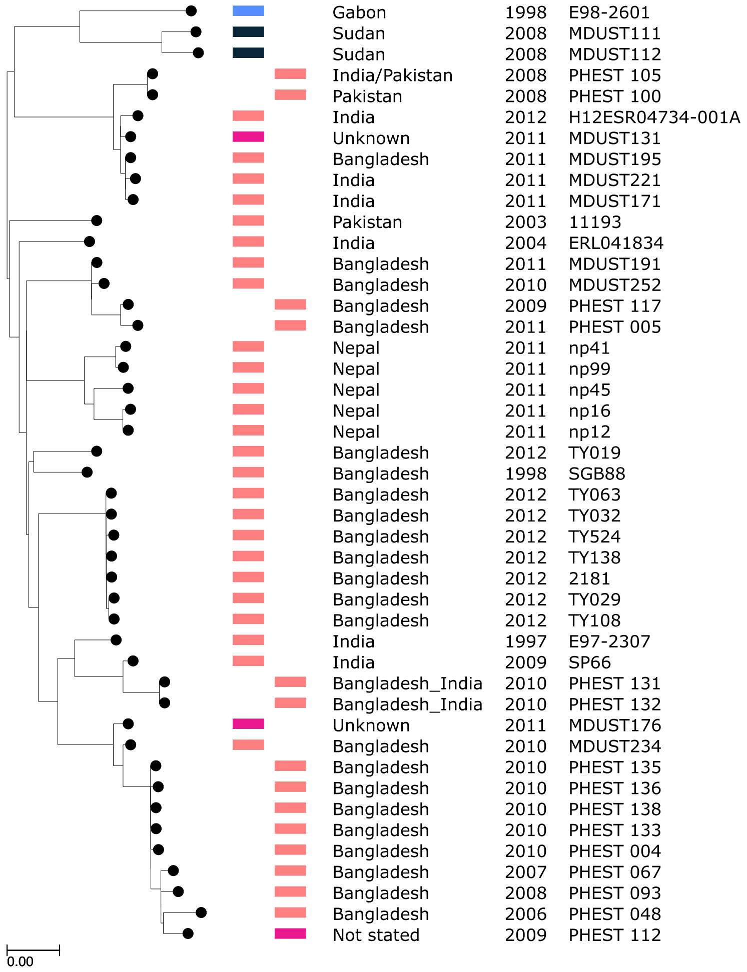

(d) Subclade 3.2.2

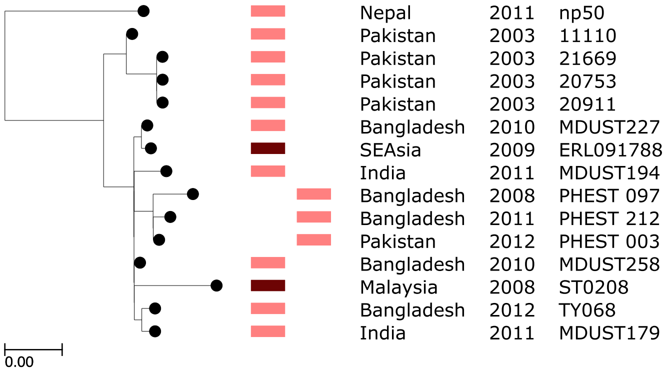

(e) Subclade 3.1.1

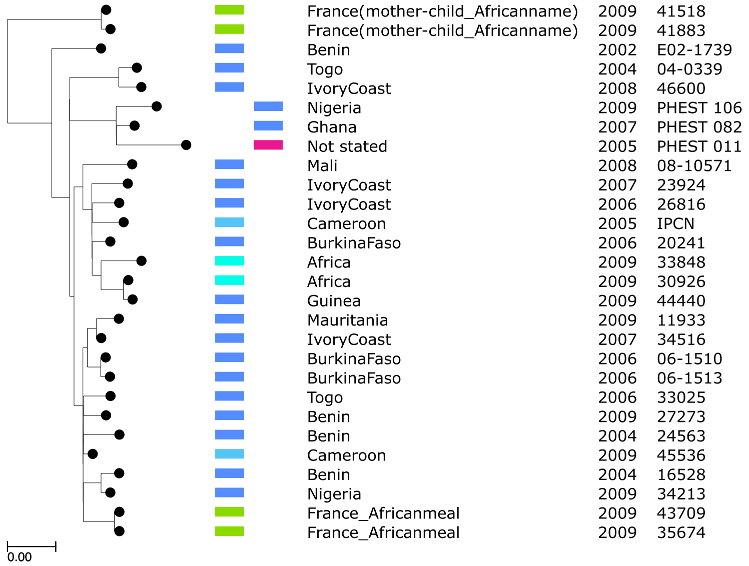

(f) Subclade 3.0.1

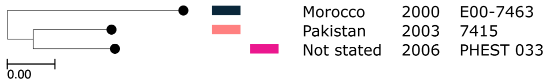

(g) Subclade 2.3.3

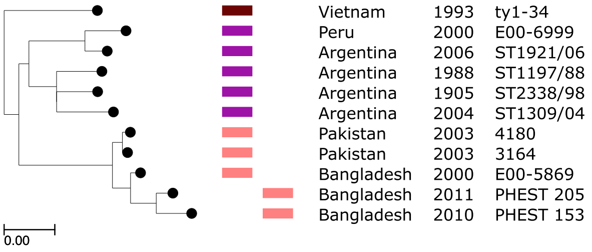

(h) Subclade 2.2.2

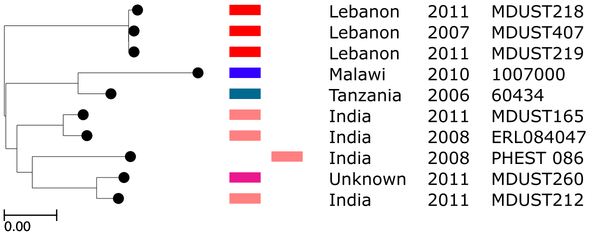

(i) Genotype 2.2.0

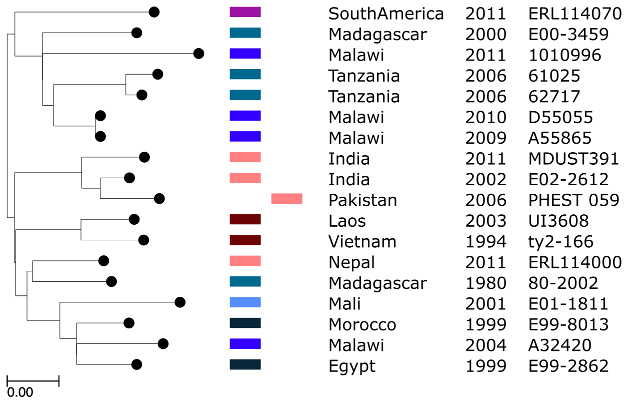

(j) Subclade 2.1.7

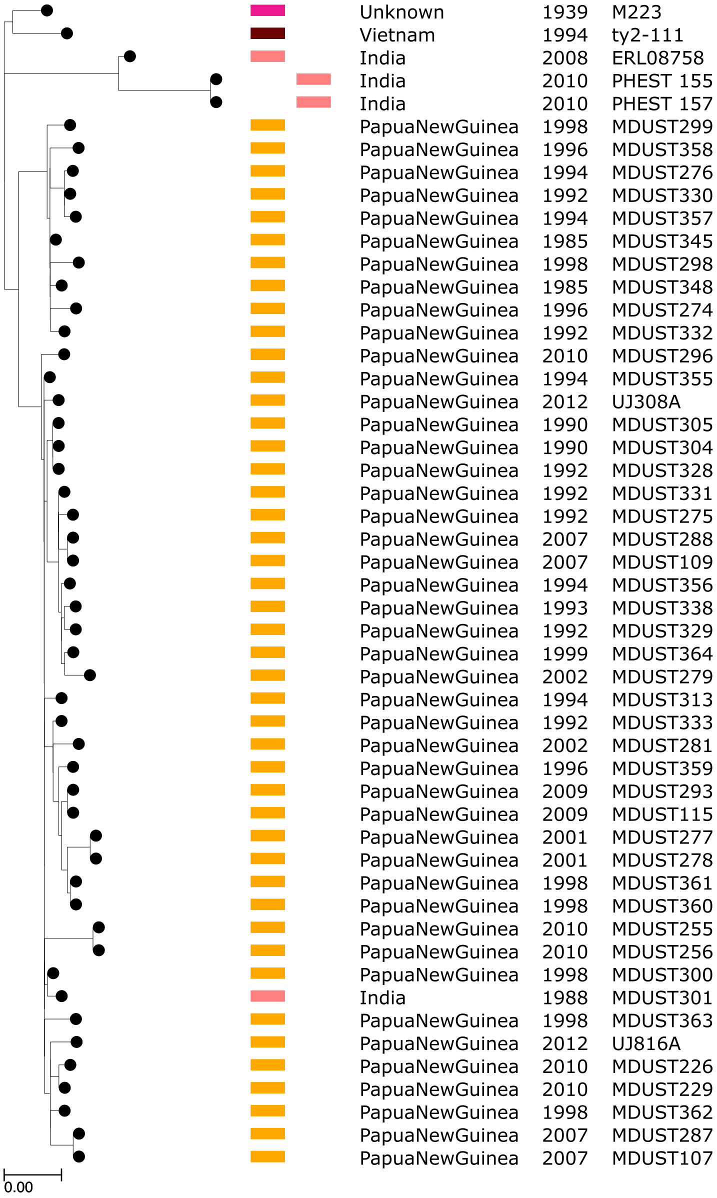

(k) Subclade 2.0.2

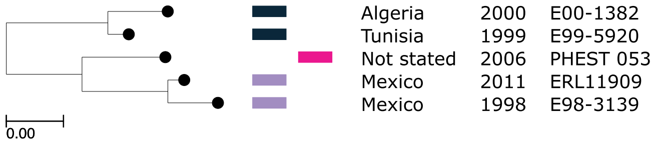

(l) Subclade 2.0.1

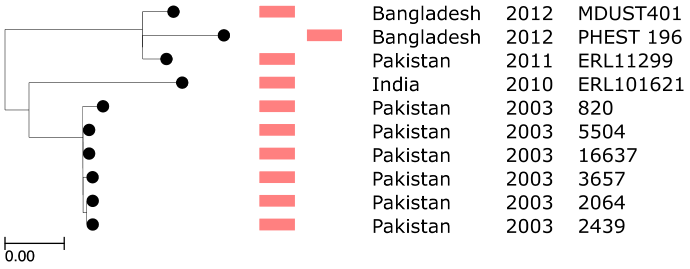

(m) Subclade 4.3.1 – singleton isolate

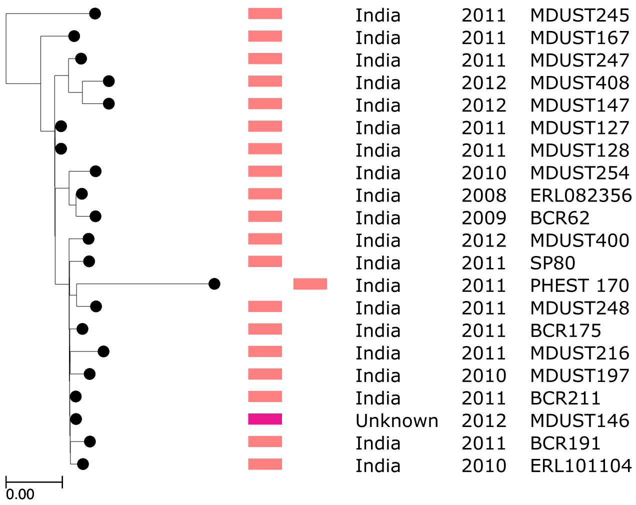

(n) Subclade 4.3.1 – travel-associated isolates subgroup

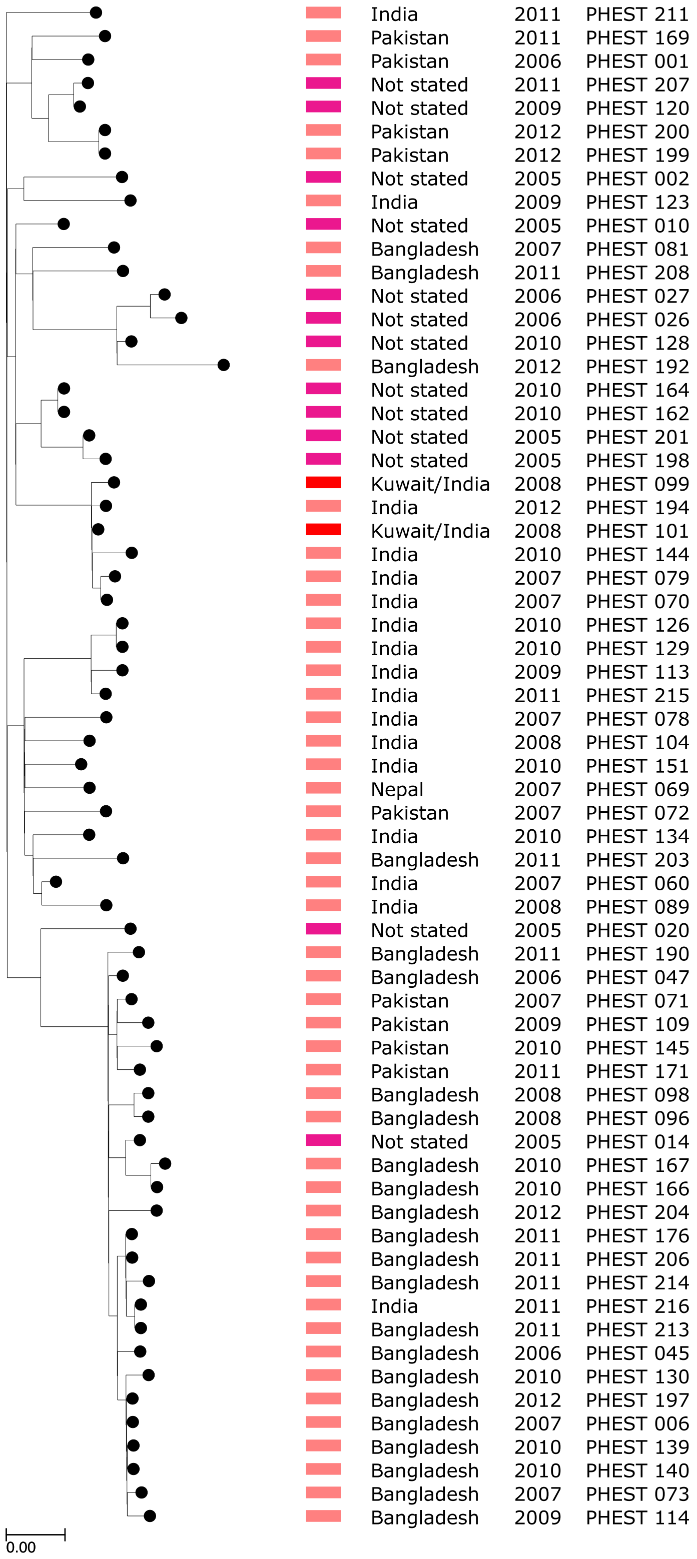

Supplementary Figure 4. Phylogenetic tress for subclades with travel-associated *S.* Typhi isolates. Each subclade with travel-associated isolates is shown (a) to (n). Tree backbone showing *S.* Typhi isolates labeled by colored bars with respect to country of origin from our global collection (first column) and travel-associated isolates (second column). For each isolate the country (third column),

year (fourth column) of isolation, and original laboratory number (fifth column) are also indicated.

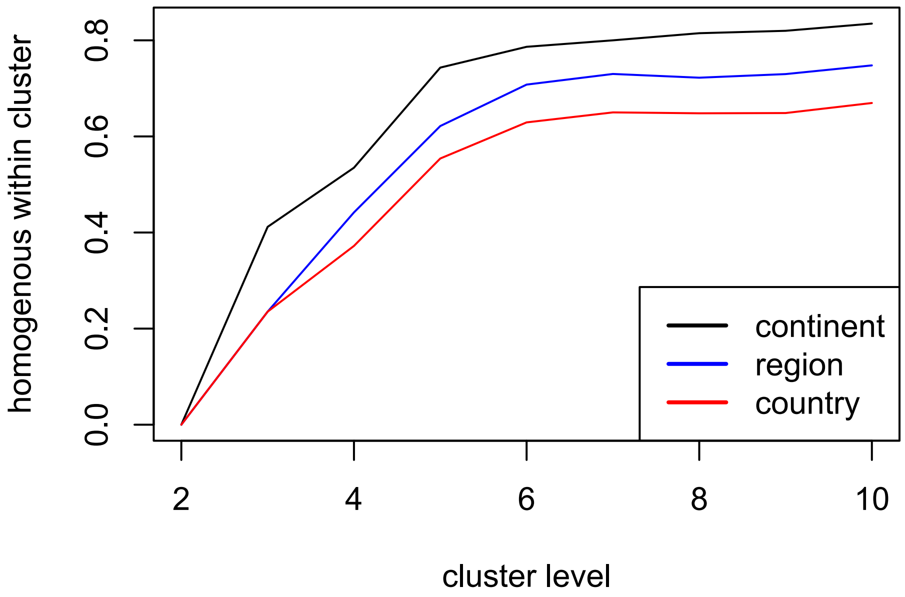

Supplementary Figure 5. Homogeneity within Bayesian Analysis of Population Structure (BAPS) clusters. Analysis of the overall homogeneity within each of the defined BAPS clusters (2-10), using the Simpson diversity index, for continent, region within continent and country.

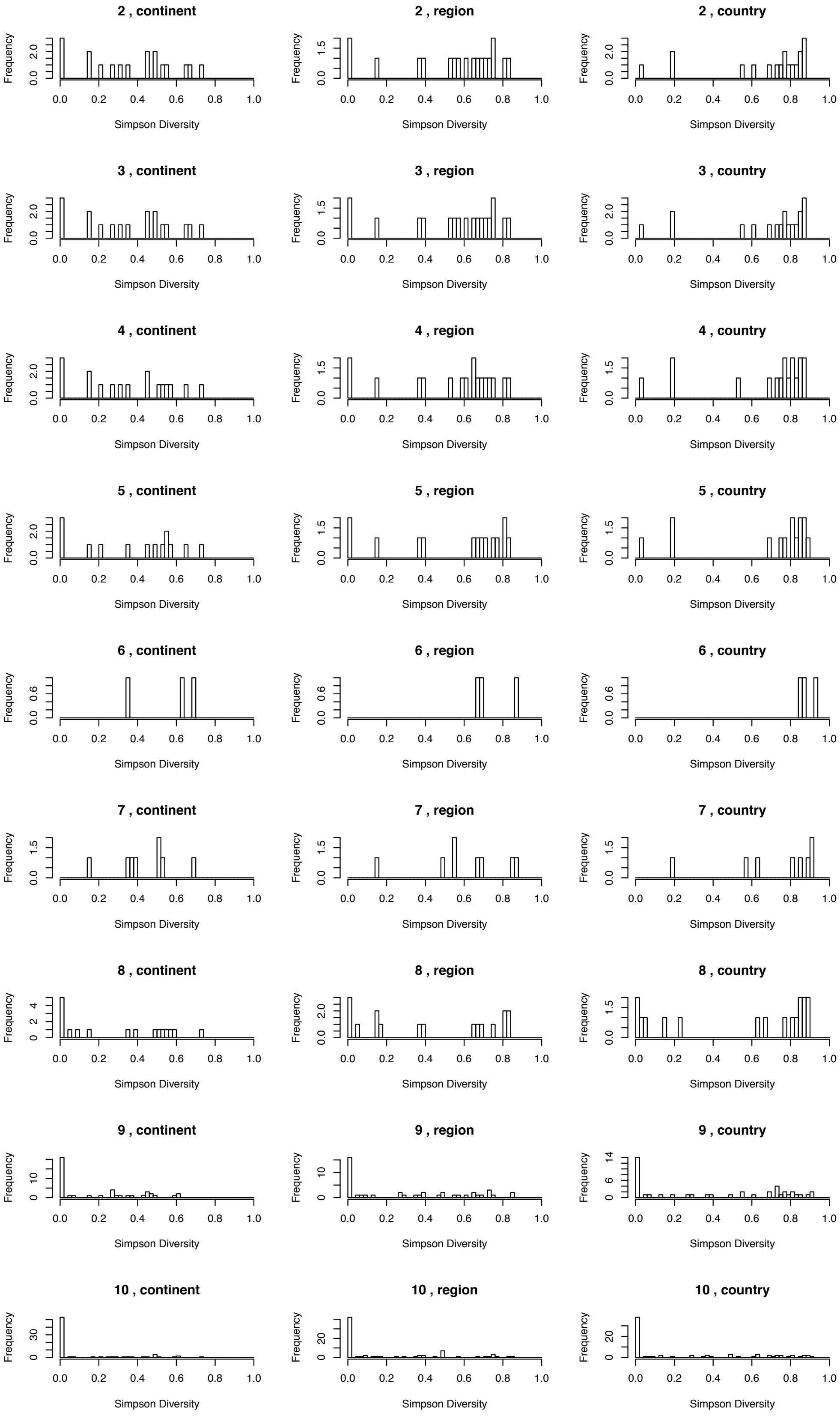

Supplementary Figure 6. Histogram distribution of homogeneity within Bayesian Analysis of Population Structure (BAPS) clusters. Distribution of the homogeneity within each of the defined BAPS clusters (2-10), using the Simpson diversity index, for continent, region within continent and country.

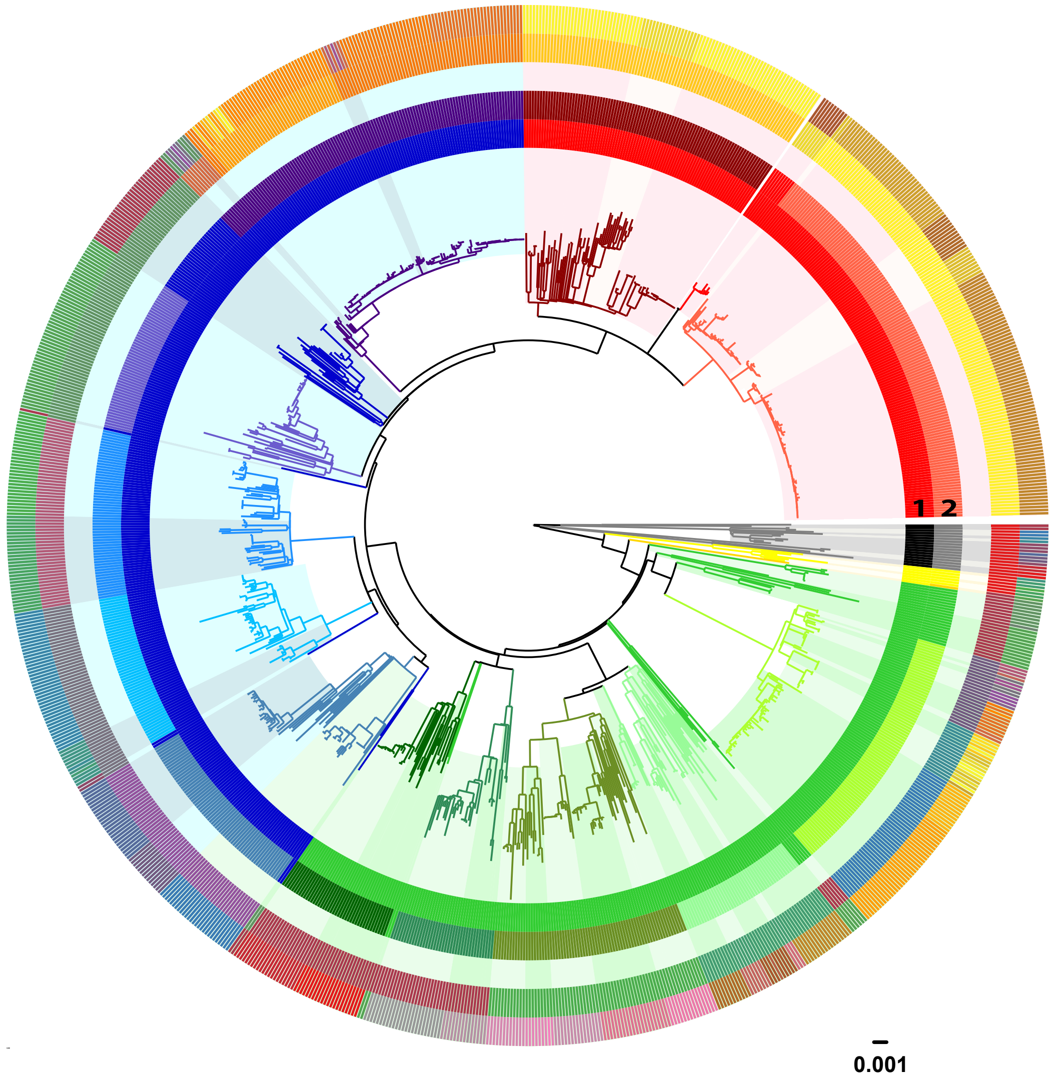

Supplementary Figure 7. Global *S.* Typhi tree showing the final genotype structure (primary clusters, clades and subclades) and raw BAPS clusters. Tree shows maximum likelihood tree of 1,831 *S.* Typhi as in **Figure 1**. Branches are colored by clade. Colored rings indicate: **(1)** Primary clusters (as labeled); **(2)** Clades (as labeled); **(3)** Subclades (alternating colors within clades); **(4)** BAPS level 3 clusters (randomly assigned colors); and **(5)** BAPS level 6 clusters (randomly assigned colors).

Supplementary Data 1. Isolates used in this study (see excel file)

Supplementary Table 1.

(a) Public reference strains of *S.* Typhi

Isolate name	Accession number	Year of isolation	Continent	Region within continent	Country
BL196	SalTypBL196v1	2005	Asia	Southeast Asia	Malaysia
CR0044	SalTypSTCR0044v1	2007	Asia	Southeast Asia	Malaysia
CR0063	SalTypCR0063 v1	2007	Asia	Southeast Asia	Malaysia
P-stx-12	ASM24553v1	2012	Asia	South Asia	India
ST0208	YKP860805.1	2008	Asia	Southeast Asia	Malaysia
UJ308A	SalTypUJ308Av1	2012	Australia & Oceania	Oceania	Papua New Guinea
UJ816A	SalTypUJ816Av1	2012	Australia & Oceania	Oceania	Papua New Guinea
E98_3139*	ASM18037v1	1998	North America	North America	Mexico
J185SM*	ASM18031v1	1985	Asia	Southeast Asia	Indonesia
CT18*R	AL513382.1	1993	Asia	Southeast Asia	Vietnam
Ty2*	ERR343332	1916	Europe	Eastern Europe	Russia
404ty*	ERR343338	1983	Asia	Southeast Asia	Indonesia
E00-7866*	ERR343336	2000	Africa	North Africa	Morocco
E02-1180*	ERR343337	2002	Asia	South Asia	India
E98-0664*	ERR343334	1998	Africa	East Africa	Kenya
E98-2068*	ERR343335	1998	Asia	South Asia	Bangladesh
M223 *	ERR349340	1939	Unknown	Unknown	Unknown
150(98)S*	ERR357622	1998	Asia	Southeast Asia	Vietnam
8(04)N*	ERR343343	2004	Asia	Southeast Asia	Vietnam
E02-2759*	ERR343339	2002	Asia	South Asia	India
E03-4983*	ERR352601	2003	Asia	Southeast Asia	Indonesia

(b) Public reference plasmids and phages

**IncHI1 plasmids**	**Accession number**
R27	AF250878
pHCM1	AL513383
pAKU1	AM412236
**Other plasmids**	**Accession number**
pHCM2	NC_003385.1

Supplementary Table 1 (a) Public reference strains used in this study (b) Public reference plasmids and phages. Key: *= *S.* Typhi isolates used in study by Holt, K. E. *et al.* (2008) [2]. R = Reference strain used in chromosomal phylogenetic analyses.

Supplementary Table 2.

Group	N	In	Out	CT18 position	Gene	Product	Nt	Codon	ns	s	%Div
**0.1**	11	T	C	655112	STY0653	alkyl hydroperoxide reductase c22 protein	547	183	0	2	0.35
**0.0.1**	2	A	G	773487	STY0776	succinate dehydrogenase hydrophobic membrane anchor protein	333	111	0	1	0.29
**0.0.2**	2	C	T	1804415	STY1910	putative ATP/GTP-binding protein	996	332	0	3	0.27
**0.0.3**	2	A	G	1840727	STY1951	putative ATP-dependent helicase	1302	434	1	6	0.37
**0.1.1**	2	A	G	3640678	STY3775	primosomal protein replication factor	825	275	0	10	0.46
**0.1.2**	1	T	C	270120	STY0255	acetyl-coenzyme A carboxylase carboxyl transferase subunit alpha	196	66	0	1	0.10
**0.1.3**	1	A	G	102135	STY0104	CorD protein	330	110	0	1	0.26
**1**	7	C	T	316489	STY0304	conserved hypothetical protein	117	39	14	6	1.49
**1.1**	6	T	C	4105384	STY4239	conserved hypothetical protein	267	89	0	1	0.17
**1.1.1**	1	A	C	555826	STY0548	putative ABC transporter ATP-binding protein	117	39	0	2	0.30
**1.1.2**	2	T	C	2360997	STY2526	Ais protein	273	91	2	5	1.15
**1.1.3**	2	T	C	4664137	STY4803	ornithine carbamoyltransferase	213	71	11	13	2.39
**1.1.4**	1	A	G	2166082	STY2335	uridine kinase	81	27	0	1	0.16
**1.2**	1	T	C	30192	STY0031	fimbrial subunit	195	65	0	1	0.19
**1.2.1**	1	T	C	4288272	STY4419	putative lipoprotein	588	196	0	2	0.31
**2**	319	A	G	2737027	STY2867	hypothetical protein	171	57	1	3	0.72
**2.0.1**	9	G	T	1215983	STY1260	putative ROK-family protein	651	217	2	2	0.44
**2.0.2**	4	A	G	4132985	STY4268	gluconate utilization operon repressor	237	79	3	3	0.60
**2.1**		A	G	146673	STY0144	UDP-N-acetylmuramoylalanyl-D-glutamyl-2,6-diami nopimelate--D-alan alanyl ligase	297	99	0	4	0.29
**2.1.1**	3	A	G	2517324	STY2682	putative oxidoreductase	283	95	0	3	0.38
**2.1.2**	2	T	C	3920009	STY4059	conserved hypothetical protein	768	256	2	5	0.80
**2.1.3**	3	A	G	3276735	STY3432	D-galactarate dehydratase	909	303	0	1	0.06
**2.1.4**	1	T	C	1173984	STY1220	flagellar P-ring protein precursor	360	120	2	4	0.54
**2.1.5**	2	A	G	2683312	STY2819	putative oxidoreductase	168	56	4	4	0.87
**2.1.6**	9	A	G	4013386	STY4154	putative DNA-binding protein	73	25	1	3	0.98
**2.1.7**	49	A	G	4094437	STY4227	nickel responsive regulator	222	74	0	1	0.25
**2.1.8**	6	A	G	827036	STY0829	biotin synthesis protein BioC	93	31	1	1	0.26
**2.1.9**	12	T	C	3476114	STY3622	uroporphyrinogen III synthase	195	65	3	3	0.81
**2.2**		C	T	4355243	STY4468	lysR family regulatory protein	156	52	0	3	0.34
**2.2.1**	11	A	G	2723847	STY2853	putative sigma(54) modulation protein	300	100	0	2	0.59
**2.2.2**	9	C	T	4388609	STY4497	alpha-galactosidase	1092	364	3	2	0.37
**2.2.3**	7	A	G	703762	STY0707	glutamate/aspartate	255	85	0	4	0.55

						transport ATP-binding protein GltL				
**2.2.4**	4	A	G	431216	STY0419	pyrroline-5-carboxylate reductase	351	117	0	2	0.25
**2.3**	72	A	G	3095443	STY3228	D-erythrose 4-phosphate dehydrogenase	717	239	2	7	0.86
**2.3.1**[a]	21	T	C	316186	STY0303	probable lipoprotein	354	118	0	2	0.37
**2.3.2**	15	T	C	1934711	STY2080	putative cation transporter	468	156	1	2	0.34
**2.3.3**	9	T	C	2811222	STY2937	glycine betaine-binding periplasmic protein precursor	48	16	2	1	0.30
**2.3.4**	12	T	C	3092900	STY3226	fructose 1,6-bisphosphate aldolase	927	309	1	3	0.37
**2.3.5**[b]	15	G	A	2723724	STY2853	putative sigma(54) modulation protein	177	59	0	2	0.59
**2.4**	38	C	A	3437570	STY3584	sec-independent protein translocase protein	621	207	0	3	0.389
**2.4.1**	24	A	G	1780319	STY1876	hypothetical protein	126	42	0	1	0.57
**2.5**	42	T	C	1535365	STY1588	pyridine nucleotide transhydrogenase subunit-beta	342	114	4	5	0.65
**2.5.1**	21	T	C	1792810	STY1897	2-dehydro-3-deoxyphosphooctonate aldolase	54	18	0	2	0.23
**3**[c]	399	T	C	3062270	STY3196	lysyl tRNA synthetase (LysRS)	989	323	3	4	0.46
**3.0.1**	2	C	T	1799842	STY1906	ribose-phosphate pyrophosphokinase	480	160	0	4	0.42
**3.0.2**	2	T	C	432732	STY0421	shikimate kinase II	387	129	0	2	0.37
**3.1**	66	G	A	3069182	STY3203	conserved hypothetical protein	9	3	0	1	0.37
**3.1.1**	25	A	G	2732615	STY2863	30S ribosomal subunit protein S16	154	52	1	3	1.60
**3.1.2**	27	T	C	3770391	STY3909	ATP synthase subunit B	177	59	0	2	0.42
**3.2**	58	A	G	2269835	STY2438	endonuclease IV	819	273	2	3	0.58
**3.2.1**	44	T	C	4215341	STY4333	phosphoribulokinase	294	98	1	3	0.46
**3.2.2**	12	T	C	4602946	STY4741	probable sugar phosphotransferase	444	148	1	2	0.65
**3.3**	62	T	C	3368641	STY3527	conserved hypothetical protein	177	59	2	2	0.99
**3.3.1**	32	A	G	2245432	STY2413	cytidine deaminase	684	228	1	2	0.34
**3.4**	56	A	C	3164162	STY3316	hydrogenase-2 component protein	30	10	1	2	0.61
**3.5**	122	A	G	3923165	STY4063	deoxyuridine 5'-triphosphate nucleotidohydrolase	411	137	0	1	0.22
**3.5.1**	1	T	C	1811809	STY1918	hydrogenase-1 operon protein HyaF	738	246	1	3	0.47
**3.5.2**	5	T	G	3729635	STY3874	glutamine synthetase	1317	439	1	2	0.21
**3.5.3**[d]	6	T	C	3817752	STY3949	hypothetical protein	586	196	4	2	0.53
**3.5.4**	31	T	C	183033	STY0176	dihydrolipoamide acetyltransferase component (E2) of pyruvate dehydrogenase	969	323	3	7	0.53
**4**	1089	T	C	1615350	STY1689	conserved hypothetical protein	105	35	1	3	1.15
**4.1**	96	A	G	2342045	STY2507	ribonucleoside-diphosphate reductase 1 beta chain	588	196	1	6	0.62
**4.1.1**	18	T	C	3996717	STY4134	alpha-amylase	1623	541	2	6	0.39
**4.2**	140	A	G	2640029	STY2781	SseB protein	210	70	2	2	0.51
**4.2.1**	40	T	C	989024	STY0994	killing factor KicB	312	104	2	3	0.38
**4.2.2**	77	A	G	3806278	STY3940	chromosomal replication initiator protein	1152	384	6	3	0.64

**4.2.3**	12	A	G	1611156	STY1683	putative oxidoreductase	502	168	1	4	0.56
**4.3.1**[e]	853	T	C	2348902	STY2513	anaerobic glycerol-3-phosphate dehydrogenase subunit A	1047	349	2	8	0.61

Supplementary Table 2. Canonical SNPs for genotyping *S.* Typhi into Clades and Subclades. Key: Group = Primary cluster/Clade/Subclade; N = number of *S.* Typhi isolates in the Group; In = allele within the group; Out = allele outside of group; Nt = nucleotide position in the gene; Codon = codon position in the gene; ns = number of non-synonymous SNPs in the gene; s = number of synonymous SNPs in the gene; %Div = % of diversity in the gene; [a] nested in 2.3.2; [b] nested in 2.3.3; [c] BiP48; [d] nested in 3.5.4; [e] BiP33.

Supplementary References

1. Roumagnac, P. *et al.* Evolutionary history of Salmonella typhi. *Science* **314**, 1301-4 (2006).
2. Holt, K.E. *et al.* High-throughput sequencing provides insights into genome variation and evolution in Salmonella Typhi. *Nat Genet* **40**, 987-93 (2008).
